# MetalHawk: Enhanced Classification of Metal Coordination Geometries by Artificial Neural Networks

**DOI:** 10.1021/acs.jcim.3c00873

**Published:** 2023-11-13

**Authors:** Gianmattia Sgueglia, Michail D. Vrettas, Marco Chino, Alfonso De Simone, Angela Lombardi

**Affiliations:** † Department of Chemical Sciences, University of Naples Federico II, Via Cintia 21, 80126 Napoli, Italy; ‡ Department of Pharmacy, University of Naples Federico II, Via Domenico Montesano 49, 80131 Napoli, Italy

## Abstract

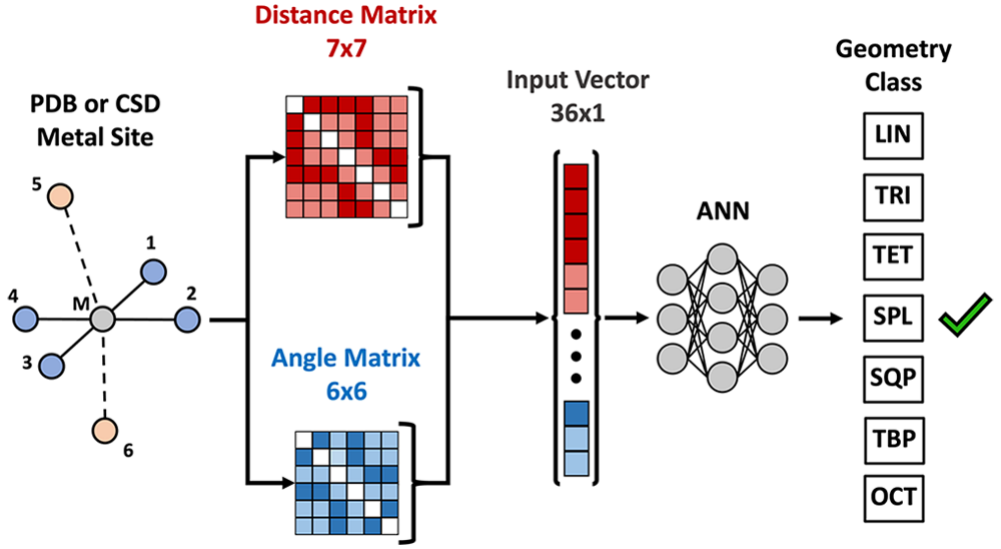

The chemical properties of metal complexes are strongly dependent on the number and geometrical arrangement of ligands coordinated to the metal center. Existing methods for determining either coordination number or geometry rely on a trade-off between accuracy and computational costs, which hinders their application to the study of large structure data sets. Here, we propose MetalHawk (https://github.com/vrettasm/MetalHawk), a machine learning-based approach to perform simultaneous classification of metal site coordination number and geometry through artificial neural networks (ANNs), which were trained using the Cambridge Structural Database (CSD) and Metal Protein Data Bank (MetalPDB). We demonstrate that the CSD-trained model can be used to classify sites belonging to the most common coordination numbers and geometry classes with balanced accuracy equal to 96.51% for CSD-deposited metal sites. The CSD-trained model was also found to be capable of classifying bioinorganic metal sites from the MetalPDB database, with balanced accuracy equal to 84.29% on the whole PDB data set and to 91.66% on manually reviewed sites in the PDB validation set. Moreover, we report evidence that the output vectors of the CSD-trained model can be considered as a proxy indicator of metal-site distortions, showing that these can be interpreted as a low-dimensional representation of subtle geometrical features present in metal site structures.

## 1 Introduction

Machine Learning (ML) is a subfield of artificial intelligence that studies algorithms able to autonomously learn from data, improving on a specific task for which they are not explicitly programmed.^1,2^ ML has become increasingly prevalent in almost all aspects of scientific research, mainly thanks to the ability of ML models to learn patterns and relationships from the training data, which makes them uniquely suited to tackle complex problems.^3−7^ The use of machine learning for the study and discovery of new metal compounds is a growing area of research that combines the power of machine learning algorithms with the vast amount of data generated by experimental studies.^8−10^ Metal sites provide the structural and chemical basis for a variety of activities and properties displayed by molecular complexes, metalloproteins, metal–organic frameworks, and other metal-based compounds.^11−14^ Most known catalysts generally contain metal ions as the heart of their reactivity. Said reactivity is strongly dependent on several site features like metal and ligand identity, coordination number, coordination geometry, long-range interactions, and more subtle chemical properties. The same can be said for metalloproteins, with additional considerations arising from the inclusion of metal centers in intrinsically more complex systems as in the case of polypeptides and other biomolecules,^15,16^ for which several specialized ML-based tools have been developed recently.^17−24^

Ultimately, the goal of applying ML models to the study of metal sites is to identify patterns in the data and make predictions about the properties of new metal compounds, which can then be synthesized and studied experimentally. In a supervised learning framework, ML models rely on annotation of molecular features for a number of structures as large as possible. Manual inspection of the structures and annotation of geometric properties of interest is generally unfeasible, even for databases of modest size, making the development of automated methods for molecular property classification an indispensable precondition for training more advanced ML models devoted to specific applications.^25−27^ Among automated methods, ML-based algorithms have also been shown to be a valid strategy to perform molecular property classification, provided it is possible to assemble sufficient data for algorithm training. This has become progressively easier in recent times, thanks to the steady increase in the number of compounds containing metal sites in structural databases.^28−30^ Although this represents a clear advantage, especially for training more complex models, property annotation can still be a challenge in and of itself even through automated methods.

Most tools developed so far to perform metal site coordination number (CN) and geometry classifications are based on traditional approaches, which rely on a clear mathematical model, designed to assign a coordination environment based on specific parameters. Several computational strategies have been devised over the years for the determination of CN in crystal structures, including methods based on distance cutoff values,^31^ reciprocal gaps,^32^ relative distances,^33^ and Voronoi analysis-based strategies.^34,35^

Classification of coordination geometry, on the other hand, usually relies on metrics encoding how close a given structure is to the perfect polyhedra used as reference. Root mean square deviation (RMSD), continuous symmetry measures (CSMs),^36,37^ and continuous shape measures (CShMs)^38^ have been used successfully as similarity metrics for this purpose; however, their calculation can be intensive, especially for larger CNs, and it is subjected to the requirement that the CN is known.

Despite these limitations, several computational tools have been proposed for automated recognition of coordination environments using the methods outlined above. One of the earliest examples is FindGeo,^39^ a Python/Fortran program, in which metalloprotein site geometry is assigned through superposition of the examined site to ideal geometries, performed through the quaternion parametrization of rotation method. RMSD is then calculated to determine the closest structural match, and sites are tagged as *regular*, *distorted*, or *irregular* depending on the specific numerical thresholds computed from the symmetrical matrices containing the RMSD values of perfect geometries calculated from the superposition among each other.

A different approach for metal site geometry assignment has been undertaken by CheckMyMetal,^40^ a metal site validation server designed for biomolecules. Here, gRMSD, a parameter defined as the RMSD of all angles formed by two ligand atoms and having the metal atom as vertex, is computed and used to assign the best possible match by comparison to ideal polyhedra. A series of other parameters, such as the geometric deviation and penalties for vacant coordination sites and uncommon geometries, have been incorporated into a weighted scoring function.

More recently ChemEnv,^41^ a tool for automated assignment of both CN and coordination geometry, has been proposed and is included in the Pymatgen^42^ Python package. The authors devised a sophisticated approach based on a modified Voronoi analysis to determine the nearest neighbors, which are then compared to ideal polyhedra using a similarity metric based on CSM. The explosion of complexity in CSM calculation for larger CNs is also cleverly addressed by means of a plane separation algorithm. The final assignment can be performed through two different strategies, one of which confers to this approach some degree of robustness to small distortion in metal sites.

Although remarkable in their implementation, the most accurate among the aforementioned methods also tend to be the most computationally intensive, making it difficult to propose their application for classifying very large numbers of sites. Availability of annotated properties for known compounds is a necessary precondition to gain valid insight from experimentally characterized systems, either by statistical inference or by ML-based techniques and to use it to design either novel metal compounds^43,44^ or metalloproteins de novo.^45−50^

**Figure 1:**
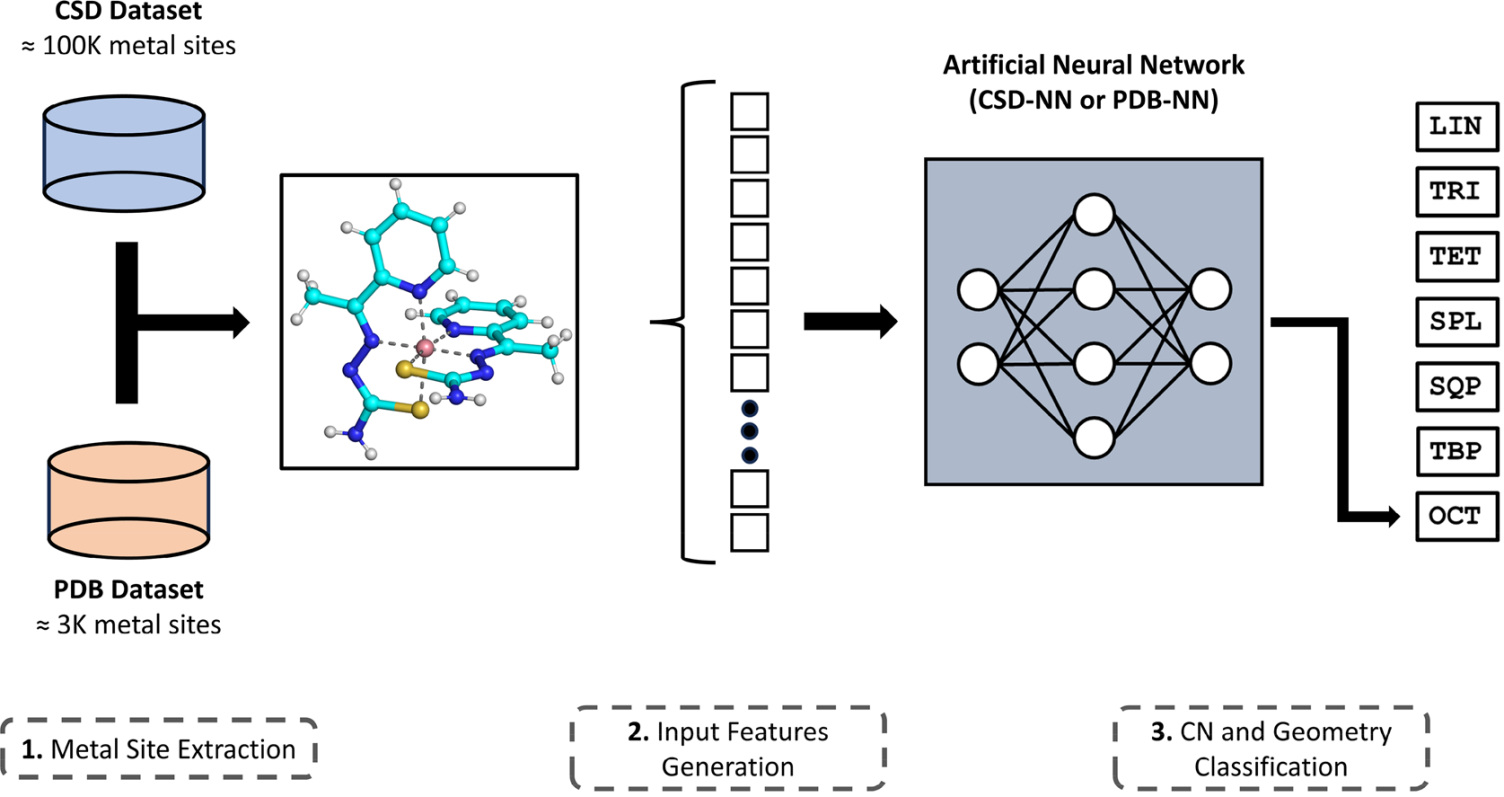
General pipeline for the ANN classifiers: 1) Metal sites are isolated from the respective crystal structures with a 10-Å radius sphere centered on the metal. 2) Geometric features including distances and angles are calculated from the Cartesian coordinates of the metal atom and its six closest neighbors. 3) Calculated features are used as input vectors to train a feed-forward ANN (*i.e*. a Multilayer Perceptron) classifier for simultaneous recognition of metal site CN and geometry.

Given these challenges, we propose a new ML approach to address the need for fast and accurate methods to computationally determine coordination number and geometry of metal sites (Figure 1). We trained and tested two different artificial neural networks (ANNs),^51^ to perform simultaneous coordination number and geometry assignment of metal sites derived from the Cambridge Structural Database (CSD)^52^ and from the Protein Data Bank (PDB).^53^ Structures containing metal sites were identified in the respective databases and used to assemble two data sets containing approximately 3,000 and 100,000 metal sites from the PDB and CSD, respectively. Metal sites isolated from the original crystal structures were subsequently processed to extract spatial characteristics to be used as input features for fully connected, feed-forward ANNs, which perform multiclass classification, assigning the metal site to the respective geometry and its implied coordination number. The two models, trained on CSD- and PDB-derived metal sites, were validated on both types of data as a further test of their performance and to offer some understanding of the spatial patterns learned by the models themselves. Finally, we used the entropy of the output probability distribution from the more robust CSD model as a proxy, to detect the metal site deviations from the ideal geometries.

## 2 Methods

### 2.1 CSD Data Set

The CSD data set was assembled from crystallographic coordinates of metal complexes obtained from the CSD (version 5.42) through the ConQuest^54^ program provided by the CCDC Software suite. General search parameters were as follows: nondisordered, no errors, not polymeric. Five queries were created, corresponding to metal CNs from 2 to 6, by specifying the desired CN for the metal in the ConQuest query building interface (“number of bonded atoms” parameter). The identity of the metal was restricted only to the most common transition elements also present in the PDB (Fe, Co, Ni, Cu, Zn, Mn, Mo), and accordingly, the ligand atoms were chosen to represent those more frequently found in metalloproteins (N, O, S, Se, Cl, Br, I), while allowing only single bonds to the metal. Once the searches were completed, retrieved structures were downloaded as Crystallographic Information Files (CIFs) for further manipulations. Using a Pymol script, all CIFs were used to build the corresponding crystal lattices (up to 10-Å away from the asymmetric unit), and a 10-Å radius sphere centered on each metal atom was then extracted and saved in PDB file format. Metal site geometry classification, needed for supervised training, was performed by a Python script, in which metal sites with all bond distances scaled to 2 Å, were compared by RMSD to computationally generated perfect sites, corresponding to the site geometries found in MetalPDB.^55^ Each metal site was superimposed to all the perfect geometries compatible with its assigned CN by the BruteForceOrderMatcher (BFOM) module in Pymatgen. The perfect geometry corresponding to the lowest RMSD was then assigned to the metal site. The classes considered in this work were the most represented in coordination chemistry: linear (LIN), trigonal planar (TRI), tetrahedral (TET), square planar (SPL), square pyramidal (SQP), trigonal bipyramidal (TBP), and octahedral (OCT) (Supporting Information, Table S1). All metal sites assigned to any other of the 21 geometries present in the MetalPDB database were excluded from the data set. Each of the considered classes was represented by a single integer value (0 to 6 in the order presented above), which was associated with each metal site to encode the class to which it was assigned following the classification procedure. After removal of those spheres containing interatomic distances below 1.0 Å, the full CSD data set contained 109,001 files. The CSD validation set was assembled using the same procedure but from crystal structures deposited in a newer version of the CSD (version 5.43) and excluding all sites already present in the training set, for a total of 1,369 metal sites.

### 2.2 PDB Data Set

The PDB data set was created from metalloprotein crystal structures deposited in the PDB (www.rcsb.org). Full lists of PDB codes and metal site identifiers for each of the seven classes considered were retrieved from MetalPDB (https://metalpdb.cerm.unifi.it, PDB entries deposited up to 01/01/2018) using the online advanced search tool. Only structures with resolution below 2.5 Å were considered; only sites containing any of the transition metals were kept, while those containing alkaline and alkaline-earth metals were discarded. All metal sites present in each list were then extracted from the corresponding crystal structures as 10-Å radius spheres centered on the metal, analogous to that described for the CSD data set creation. Whenever present, alternative conformations were split into different spheres, and only those in which metal site geometry was retained were kept in the data set, up to a maximum of two conformations per structure. The geometry assigned by MetalPDB was then verified using the same RMSD-based classification procedure used for metal complexes from the CSD. All classified spheres from the PDB were assembled to form the full data set, which included 2,960 files. In order to build a validation set for biomolecular sites, structures of metalloproteins deposited in the PDB after 1 January 2019 were retrieved directly from the PDB. An initial classification of coordination number was performed by using a simple cutoff criterion, considering the CN equal to the number of heteroatoms (any atom except for carbon and hydrogen) within a 2.8-Å distance of the metal atom. After assigning the CN with this simplified method, the geometry of all metal sites was determined using the RMSD-based procedure. Classified sites were then manually reviewed to verify the agreement between the true class, as determined by the authors, and the class assigned *in silico*. This led to a validation set composed of 109 metal sites in total.

### 2.3 Input Features Generation

The geometry of a metal site is encoded into the spatial coordinates of the metal atom and those of the atoms bonded to it. Given that the maximum CN in this work is six, only the six nearest ligand atoms were considered for input feature generation. To train a classification model, the use of a representation of the spatial relationship between the atoms independent of the particular coordinate system is preferable, as previously shown for various applications. The six non-hydrogen atoms closest to the metal center were identified and ordered according to their distance from the metal. The interatomic distance matrix comprising the metal atom plus the six nearest neighbors was then calculated. The upper triangular matrix elements were flattened giving a 21-value distance vector. In addition to the distances of the 6 nearest neighbor atoms from the metal (ligand–metal or L-M distances) and the ligand distances from each other (ligand–ligand or L-L distances), the 15 unique L-M-L angles (ligand–metal–ligand angles) formed by all distinct atom pairs belonging to the six atoms closest to the metal and having the metal atom itself as vertex were also calculated from the sphere files and used as input features. The input vector was then completed by addition of the L-M-L angles, giving a 36-value vector composed of both distances and angles.

### 2.4 Model Setup and Evaluation

The ANNs discussed here were constructed using the MLPClassifier implementation of the scikit-learn library^56^ (version 1.2.0). To obtain the best hyperparameter values of trained models, Bayesian hyperparameter optimization was performed using the scikit-optimize package (version 0.8.1) with the BayesSearchCV method.

Both PDB and CSD data sets were split randomly so that approximately 90% could be used for training and the remaining 10% for testing. BayesSearchCV was performed on each training set separately, optimizing for the Balanced Accuracy (BA) score. A 5-fold cross-validation was performed on the training set by BayesSearchCV for each new configuration in the optimization process. After completion of hyperparameter optimization, the best model was refitted with the entire data set, and a final 5-fold cross-validation of the best estimator was performed on the complete training sets (Supporting Information, Table S2).

Once the optimal models, one for PDB-NN and one for CSD-NN, had been obtained, they were evaluated on the 10% hold out test data sets (Table 1 and Table 2, respectively).

**Table 1:** Performance Metrics on the Test Set for the Hyperparameter Optimized CSD-NN Model and Support (Number of Metal Sites) for Each Class

Class	Precision	Recall	F1-score	Support
LIN	1.0000	1.0000	1.0000	97
TRI	1.0000	1.0000	1.0000	117
TET	0.9966	0.9932	0.9949	585
SPL	0.9752	0.9672	0.9711	1096
SQP	0.9654	0.9728	0.9691	1952
TBP	0.9709	0.9648	0.9679	796
OCT	0.9949	0.9950	0.9950	6257
Macro Average	0.9861	0.9847	0.9854	
Weighted Average	0.9861	0.9861	0.9861	10900

**Table 2:** Performance Metrics on the Test Set for the Hyperparameter Optimized PDB-NN Model and Support (Number of Metal Sites) for Each Class

Class	Precision	Recall	F1-score	Support
LIN	0.8333	0.8333	0.8333	6
TRI	0.6667	1.0000	0.8000	2
TET	1.0000	0.9923	0.9961	130
SPL	1.0000	0.8571	0.9231	7
SQP	0.7619	0.8889	0.8205	18
TBP	1.0000	0.8462	0.9167	13
OCT	0.9833	0.9833	0.9833	120
Macro Average	0.8922	0.9145	0.8962	
Weighted Average	0.9731	0.9696	0.9704	296

Finally, the optimal models were also tested on validation sets assembled from independent data not present in any of the data sets used for training and testing, assembled as previously described, and the performance metrics were calculated using the Python packages described above.

### 2.5 Entropy Calculation and Metal Site Distortion

Shannon entropy values^57^ were calculated from the output probability vectors using the Numpy^58^ package. Distortion trajectories were simulated starting from metal sites belonging to the TET, SPL, SQP, and TBP classes, taken from the PDB and CSD validation sets.

Ligand atom coordinates were rotated around the metal atom, gradually bringing the L-M-L angles closer to the values defined for the target geometry. Rotation of ligand atoms was performed keeping all L-M distances unaltered (Supporting Information, Section SI 1, Figure S1). Calculation of RMSD was performed as described above for data set classification, while calculation of Continuous Shape Measures (CShMs) was done using the cosymlib package.^59^ The effect of metal site distortion on entropy was visualized as heatmaps (Supporting Information, Figure S2) and by showing entropy distributions for different intervals of the CShM ratio (Supporting Information, Figure S3).

## 3 Results and Discussion

### 3.1 Comparison of the CSD and PDB Data Sets

**Figure 2:**
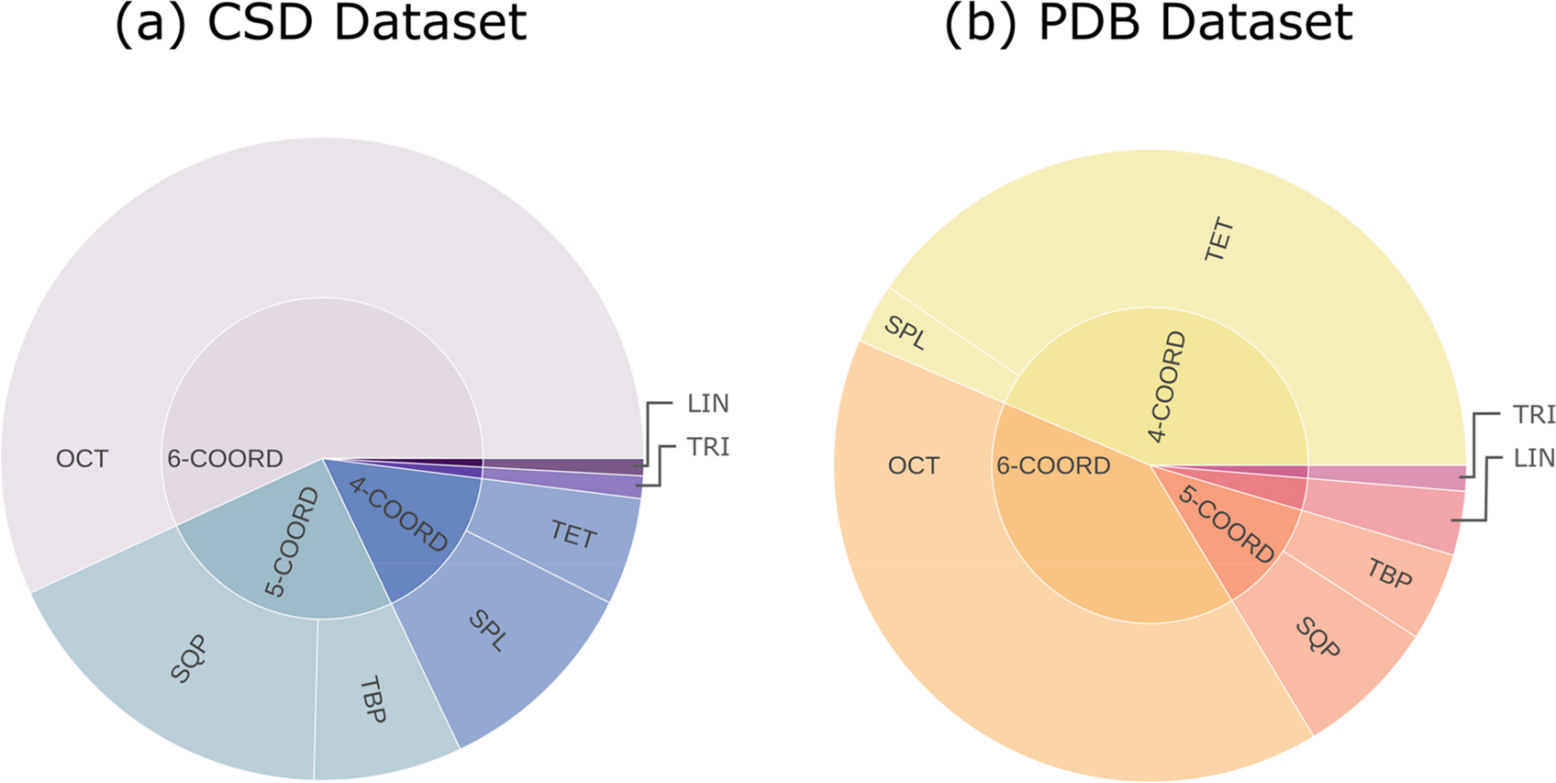
Distribution of coordination numbers (CNs) and geometries for the CSD data set (a) and (b) the PDB data set.

In this investigation, we generated data sets based on both CSD and PDB structures. These data sets are imbalanced, as they contain different classes of metal coordination that are unequally distributed (Figure 2). The most relevant difference between the two data sets is the total number of metal sites that they comprise. Whereas the CSD data set includes more than 100,000 metal sites, the PDB data set only contains slightly less than 3,000 sites.

In both sets, 6-coordinated metal sites represent the most populated class (56.88% of the CSD data set and 40.10% of the PDB data set), as expected from CN preferences for transition metals. The relative prevalence of 4- and 5-coordinated sites is instead different in the two data sets, with the CSD data set being comprised of 25.18% and 15.94% of 4- and 5-coordinated sites, respectively, and the PDB data set being comprised of 43.54% and 11.76% of 4- and 5-coordinated sites, respectively. In addition, 2- and 3-coordinated sites represent a very small minority in both data sets, as together they comprise only about 2% and 4.5% of all files in the CSD and PDB data sets, respectively.

The differences found in the two data sets are also reflected in the distributions of geometries. For the LIN, TRI, and OCT classes, the prevalence in the two data sets is equal to those mentioned for the respective CNs, since these classes only contain one possible geometry. 4- and 5-coordinated sites instead have two possible geometries each, whose populations show markedly different distributions in the two data sets. In particular, the TET and SPL geometries (CN = 4) represent 5.41% and 10.43% of the metal sites found in our CSD data set, respectively, whereas in the PDB data set, they correspond to 40.47% and 3.07%. Moreover, SQP and TBP represent 17.70% and 7.40% of the metal sites in the CSD data set, respectively, and 7.23% and 4.53% of the metal sites in the PDB data set.

In addition to a different distribution of geometries, the classes of the two data sets also present unequal distribution of metal atom identity, with individual metals clearly found to be more commonly associated with specific geometries (Supporting Information, Figures S5–S6). Metal identity distribution per class is also significantly different between the CSD and PDB data sets, partly due to the presence of more transition metals included in the PDB. Some geometries show a comparable distribution of metal site identity despite the different metal composition of the data sets, such as TRI and TET, which are mostly comprised of Cu and Zn sites, respectively. However, other geometries show some significant differences, as the SPL class, which includes almost equally Cu and Ni sites in the CSD but has a higher Ni abundance than Cu in the PDB. Similarly, the SQP geometry includes more Cu sites than Zn sites in the CSD, while in the PDB, this asymmetric distribution is reversed.

The distributions of scaled RMSD values, derived from the perfect polyhedra corresponding to the assigned class after scaling all bond distances to 2 Å, exhibit some variations between the two data sets (Supporting Information, Figure S7). This suggests that sites belonging to the CSD may be different from those belonging to the PDB, as a consequence of the different chemical composition (inorganic/organic complexes versus biomolecular sites) or to putative other biases in the data sets.

### 3.2 Model Performance

Our initial approach assumed that the spatial distribution of the atoms belonging to the first coordination sphere of the metals and their immediate neighbors would be the most important for geometry recognition. For this reason, we included in the input feature calculation only the six nearest neighbors of the metal atom, a number equal to the highest CN considered.

Using the 36-valued vectors [21 distances +15 angles] as input features, we trained two ANN classifiers, one on the CSD data set (CSD-NN) and the other on the PDB data set (PDB-NN). Balanced Accuracy (BA) and weighted F1-score (F1W) were used as guiding metrics for performance evaluation, in consideration of the strong class imbalance present in both data sets. The data discussed here refer to the cross-validation performance of the models on the respective test sets after hyperparameter optimization (Supporting Information, Tables S3–S4). The classification accuracy of CSD-NN is remarkably high (Table 1) reaching BA equal to 98.47% and F1W equal to 98.61%, with similar accuracy in all classes considered. The performance of PDB-NN (Table 2) is instead comparatively worse, with BA equal to 91.45% and F1W equal to 97.04%. Whereas the performance of CSD-NN was quite homogeneous across the different classes, that of PDB-NN is more variable, with some classes recognized better (TRI, TET, OCT) and others more poorly (LIN, SPL, SQP, TBP), as can be appreciated by inspecting the confusion matrices for the two models (Supporting Information, Figure S8).

**Figure 3:**
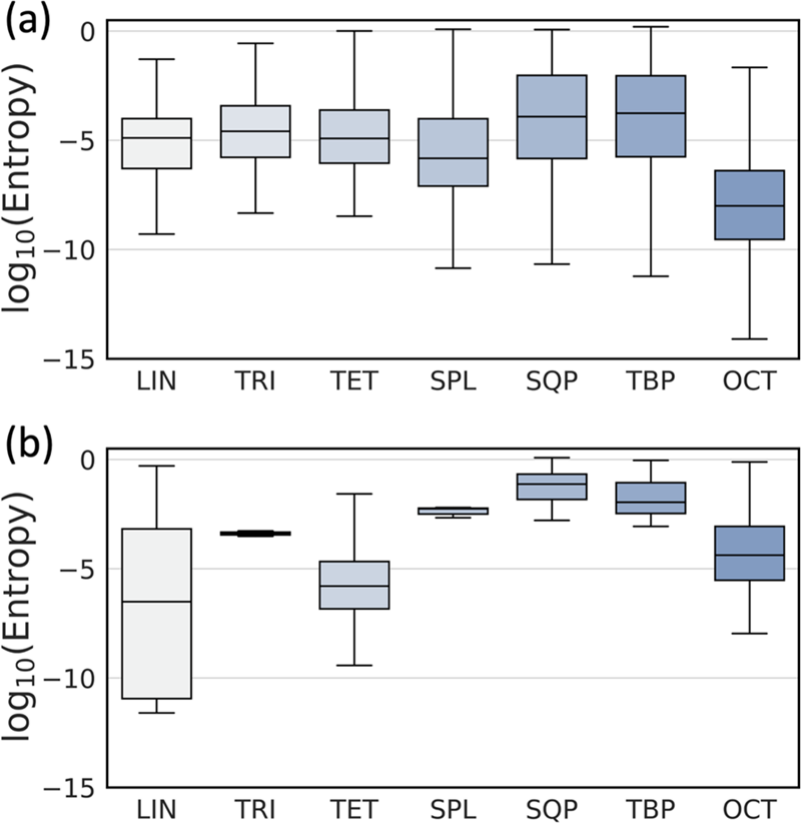
Distribution of entropy values for CSD-NN (a) and PDB-NN (b) computed on all sites in the CSD and PDB test sets, respectively.

In addition to the evaluation of classification performance, we also examined in more detail the degree of confidence displayed by the models in generating their predictions. In ANNs, the output activation layer can be interpreted as the probability that a given metal site belongs to the class encoded by the corresponding neuron. We expected a more diffuse probability distribution for sites with lower classification accuracy and a sharper probability distribution, with maximum probability for the true class, in those sites recognized more accurately. This uncertainty in class recognition, exemplified by comparable probabilities assigned to different classes, can be quantified using Shannon entropy, formally a measure of the amount of information contained in a signal. Unsurprisingly, the average entropy values for the output of the CSD-NN model are significantly lower than those of PDB-NN in 5 out of 7 classes (Figure 3 and Supporting Information, Table S5).

**Figure 4:**
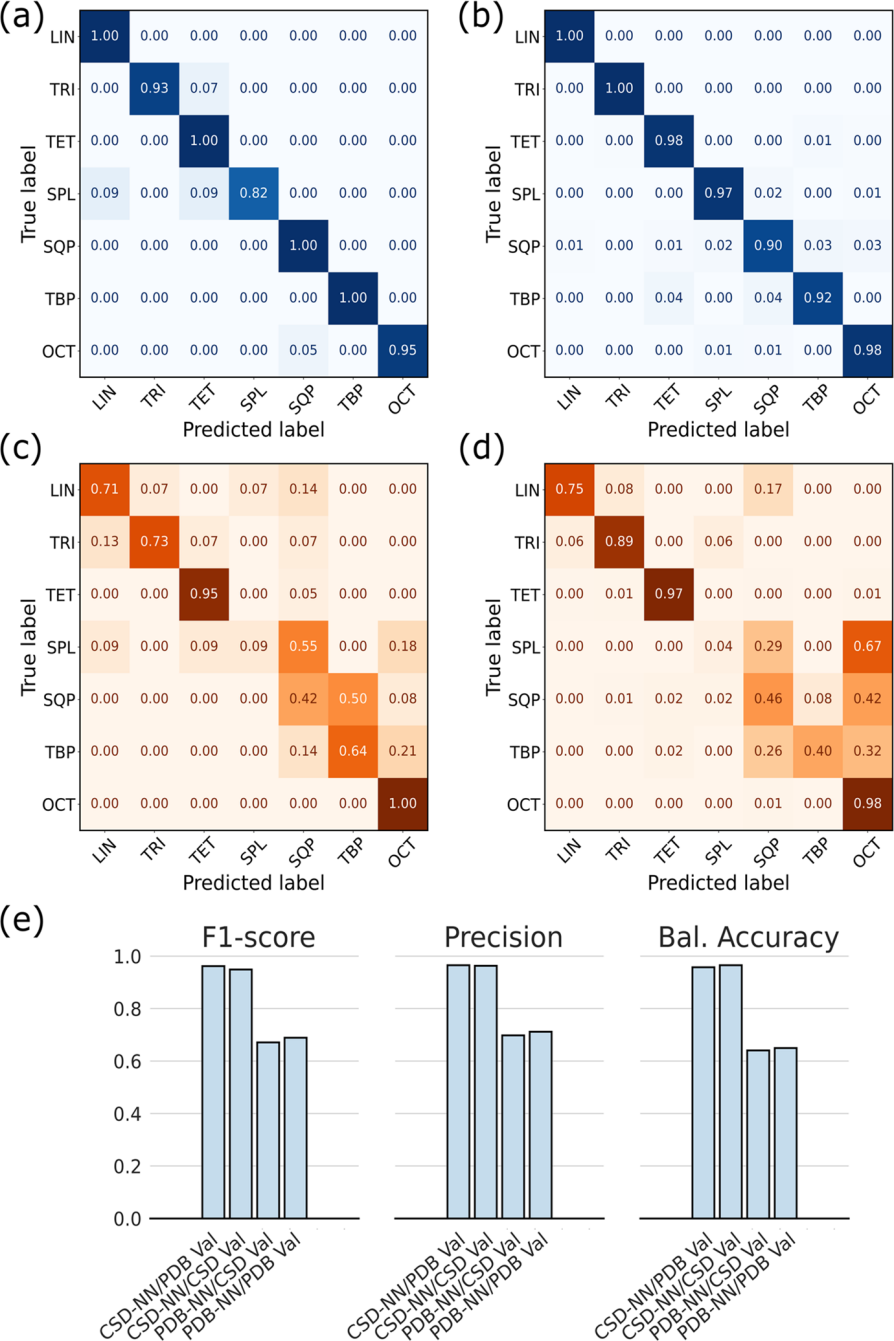
Confusion matrices detailing the performance of the CSD-NN model on the PDB validation set (a) and on the CSD validation set (b). Confusion matrices for the PDB-NN model on the PDB validation set (c) and on the CSD validation set (d). (e) Performance metrics of the CSD-NN and PDB-NN model on the validation sets. The model considered is reported before the “/”, while the validation set on which the model is tested is present immediately after and abbreviated to PDB Val and CSD Val for the PDB and CSD validation sets, respectively. The reported performance metrics are Balanced Accuracy (macro average of recalls over all classes), weighted Precision, and weighted F1-Score.

To assess more thoroughly the performance of the two models, CSD-NN and PDB-NN were tested on both the CSD and PDB validation sets (Figure 4). The two models show significantly divergent behavior in validation. CSD-NN appears to be more robust than PDB-NN, maintaining a high BA and F1W, equal to 96.51% and 96.28%, respectively, for the CSD validation set (Figure 4b, e). On the other hand, the PDB-NN shows poorer performance on the PDB validation set (BA 65.00%, F1W 67.21%), when compared to its performance on the PDB test set (Figure 4c, e). Remarkably, when the CSD-NN was tested on the PDB validation set, it retained most of its classification ability (BA 91.66% and F1W 91.33%, Figure 4a, e). Conversely the PDB-NN shows poor performance on the CSD validation set (BA 64.04% and F1W 67.11%, Figure 4d, e), very similar to that observed on the PDB validation set.

The worse performance of PDB-NN can be related to the dissimilar support for the classes in the two training data sets, which differ in both total number of metal sites and class distribution. The much larger number of sites present in the CSD data set is expected to be one of the main reasons for the comparatively better performance of the CSD-NN model. As a general trend, the most populated classes also tend to be those recognized with the highest accuracy, as in the case of TET and OCT classes. They have a significantly higher support compared to the others and are the only ones recognized quite well by the PDB-NN.

The drop in classification accuracy observed for the PDB-NN is particularly pronounced for the SPL class, which is almost always incorrectly recognized. SPL sites in the PDB data set are composed for almost 60% of third row metals Tantalum (Ta) and Platinum (Pt) (Supporting Information, Figure S6). It is possible that a high prevalence of such metals has introduced a bias for this SPL class in the PDB-NN model, compromising its ability to generalize well to more common SPL sites. Further exacerbating this issue is the fact that in the PDB validation set, the SPL class is instead composed mostly of nickel (Ni) sites, coordinated by backbone amide and histidine nitrogen atoms.

The CSD-NN model was found to perform very well on the PDB validation set (BA 91.66%, F1W 91.33%); therefore, we wondered to which extent the knowledge acquired by this model could be generally transferable to the classification of bioinorganic metal sites. In other words, we wondered if a model exclusively trained on CSD structures could be used to effectively classify metal sites from the PDB. To this end, we used the CSD-NN model to classify the entire PDB data set (Supporting InformationTable S6, Figure S9). The accuracy on the full PDB data set is significantly lower than that on the PDB validation set (BA 84.29%, F1W 93.4%), although this is mostly because of poor performance on the SPL and SQP classes. The behavior and performance of CSD-NN on PDB metal sites might be influenced by several factors. First, it should be noted that sites in the PDB validation set have been manually reviewed and therefore are expected to have a lower prevalence of abnormalities (highly distorted coordination angles and/or unusual bond distances) compared to the PDB data set. The presence of such aberrations in protein crystal structures is a documented phenomenon and can sometimes result in unrealistic metal–ligand bond lengths.^60,61^ Biomolecular crystallography is a difficult endeavor in general, and interpretation of structural data on metalloproteins poses a number of additional challenges and complexities.^62−64^ While high-resolution metalloprotein crystal structures were found to agree quite well with average metal bond lengths in the CSD database,^65,66^ medium-resolution (between 2 and 2.5 Å) structures showed significantly wider distributions, perhaps indicating the use of improperly set restraints on the metal atom position during the structure refinement protocol. Notably, even high resolution structures have sometimes been reported to contain incorrectly modeled metal sites, although much more infrequently.^67^

Indeed, we have found a number of examples of abnormal ligand–metal bond lengths in the PDB data set, sometimes approaching 2.8–2.9 Å. The presence of such abnormal metal sites in the PDB data set has undoubtedly an impact on the performance of CSD-NN, since these are expected to be much rarer among the CSD entries used to train CSD-NN. One further difference to consider is the presence in the PDB data set of transition metals from the second and third row, which are absent from the CSD data set, with the exception of Molybdenum (Mo). These are generally less abundant than lighter metals; however, there are some classes in which they constitute a large portion of all sites, most notably the LIN and SPL classes. Larger metals also tend to favor longer metal–ligand distances; however, this might not be an unsurmountable challenge for the CSD-NN model. Classification of LIN sites in the PDB data set and PDB validation set, almost half of which are Au and Hg sites, is quite accurate, while classification accuracy for the SPL class, containing mostly Pt and Ta sites, is very poor. As discussed above, the latter class contains mostly heavy metals, which are absent in the CSD data set; therefore, CSD-NN performance should be interpreted with particular caution.

In addition to differences in ligand–metal bond length distributions, RMSD distributions of the different classes are quite different between the CSD and PDB data sets (Supporting Information, Figure S7), as mentioned above. Given that RMSD values are computed after scaling all bond lengths to 2 Å, they are practically insensitive to elongated bond lengths and mostly depend on L-M-L bond angles. Despite this, notable dissimilarities still persist between the two sets, showing that different distributions of bond distances can only partially explain the CSD-NN performance on the PDB data set.

### 3.3 Entropy and Metal Site Distortion

**Figure 5:**
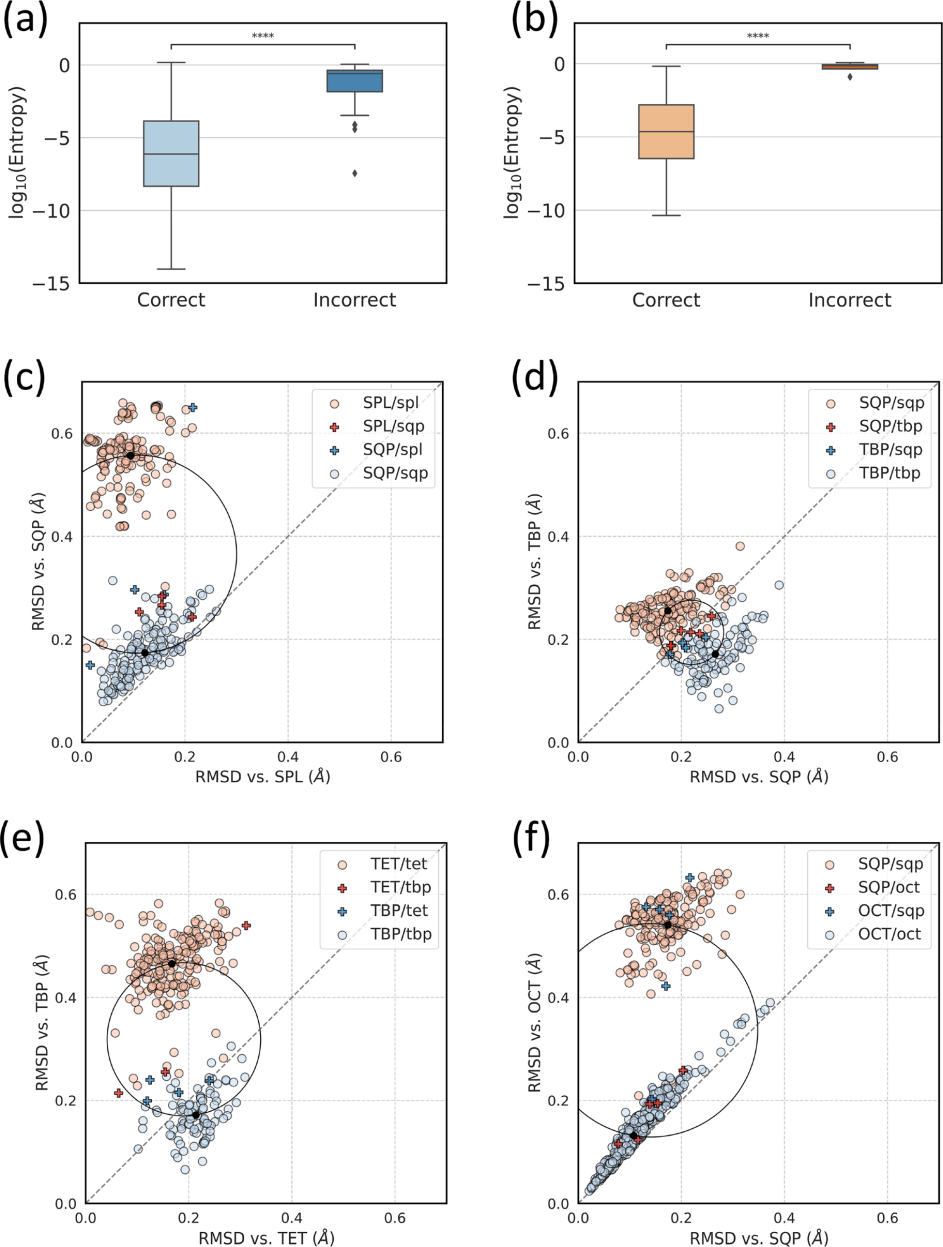
Distribution of entropy scores outputted by the CSD-NN model for correctly and incorrectly classified metal sites in the CSD (a) and PDB (b) validation sets. p-value annotation legend: ns: *p* ≤ 1.00e+00, *: 1.00e-02 < *p* ≤ 5.00e-02, **: 1.00e-03 < *p* ≤ 1.00e-02, ***: 1.00e-04 < *p* ≤ 1.00e-03, ****: *p* ≤ 1.00e-04. Black diamonds represent outliers. RMSD maps for the most common misclassifications observed for the CSD-NN model: (c) SPL/SQP, (d) SQP/TBP, (e) TET/TBP, and (f) SQP/OCT. RMSD values from sites with lower CN than the one assigned (for example SQP RMSD of an OCT site) are computed on the subset of N atoms resulting in the minimum RMSD value, among all possible subsets. In the legend, uppercase abbreviations represent the class to which a site belongs in the validation set, while the lowercase represents the class assigned by the CSD-NN model. Correctly classified sites are represented by circles, while misclassified ones are represented by crosses. “Twilight zone” between clusters is shown as black circumference, while cluster centroids are shown as black circles.

The CSD-NN model, thanks to a much larger amount of training data and a higher diversity in its composition, retains most of its classification capabilities. Despite this, some sites are also misclassified by the CSD-NN model, and these were studied more thoroughly to understand the underlying factors of this behavior. The following discussion only refers to the CSD-NN model, since the highly biased classification performance of the PDB-NN model is intrinsically more difficult to interpret. One striking difference between correctly and incorrectly classified sites is the entropy computed on the respective output vectors (Figure 5a, b). For both validation sets, the entropy of misclassified sites is significantly higher than that of correctly classified sites, indicating a higher degree of uncertainty in class assignment for the latter ones.

Based on this, we hypothesized that geometric distortions present in a metal site would impact the probabilities assigned by the model for each class, leading to a more diffuse probability distribution for sites with geometric features resembling more than one class, or in between two classes. To investigate this, sites in the validation set belonging to classes most frequently misclassified by CSD-NN were mapped using their RMSD from the real and assigned class, to elucidate whether model behavior could be attributed to geometric features present in the sites (Figure 5c-f). It should be noted that M-L distances were not scaled to a fixed length of 2 Å prior to RMSD calculation in this case, in order to fully capture the role of bond lengths in the classification and potentially detect inconsistencies in geometry and CN classification either by the CSD database or by the CSD-NN. Unexpectedly, this revealed the presence of a number of sites misclassified in the validation set that are correctly recognized by CSD-NN model. This can happen because of errors in the CN that has been assigned to a metal atom in the CSD database. It should be noted that sites for which the original classification is wrong while model classification is right still count as misclassified during performance metric calculation. Inspection of the RMSD maps also reveals that the remaining misclassified sites are often located in regions between two geometry classes, and this is particularly evident for the SQP/TBP sites. Furthermore, correctly classified sites located in this “twilight zone” situated between clusters of two different classes were found to have higher entropy compared to correctly classified sites found elsewhere on the map (Supporting Information, Figure S10). Distorted sites are seemingly more deceiving for the model, which may struggle to assign their class unequivocally, thus leading to more diffuse probability distributions in output and higher entropies. This is also partially confirmed by analysis of statistical correlation between entropy and RMSD from the corresponding ideal geometry (Supporting Information, Table S7). Moderate positive correlation of entropy with either RMSD or scaled RMSD (calculated after scaling all bond lengths to 2 Å) is found for all geometry classes with the sole exception of the TRI class in both the CSD and PDB validation sets. Similar correlations were also found between entropy and pertinent CShM values.

Finally, to further confirm the relationship between outputted probability values and geometric features present in metal sites, distortions were artificially introduced into correctly classified sites to deliberately transform SPL sites into TET sites or SQP sites into TBP sites and vice versa (Supporting Information, Figure S1). This was accomplished by rotating ligand atoms in these structures around the metal atom, progressively morphing them into the desired geometry, leaving the distance of the ligand atoms from the metal unchanged.

Distortion trajectories were constructed from the majority of metal sites belonging to both the CSD and PDB validation sets, and the degree of distortion was quantified by calculating the Continuous Shape Measures (CShMs) from the starting and final geometries. For each point of the distortion trajectory, entropy was also calculated and visualized as a heatmap (Supporting Information, Figure S2). Large scale analysis of these distortion trajectories shows in all cases that entropy is significantly higher close to the center of the trajectory (halfway between the two geometries) and for sites that are somewhat distorted either at the beginning or end of the trajectory. Conversely, the lowest entropy values are found for structure with a low CShM from one geometry and a high CShM from the other, namely more “ideal” sites. Inspection of entropy distributions for different ranges of ratio between the CShMs also confirms that the highest average entropy is found close to zero, while the lowest values are at the extremes for both CSD and PDB validation sets (Supporting Information, Figure S3).

**Figure 6:**
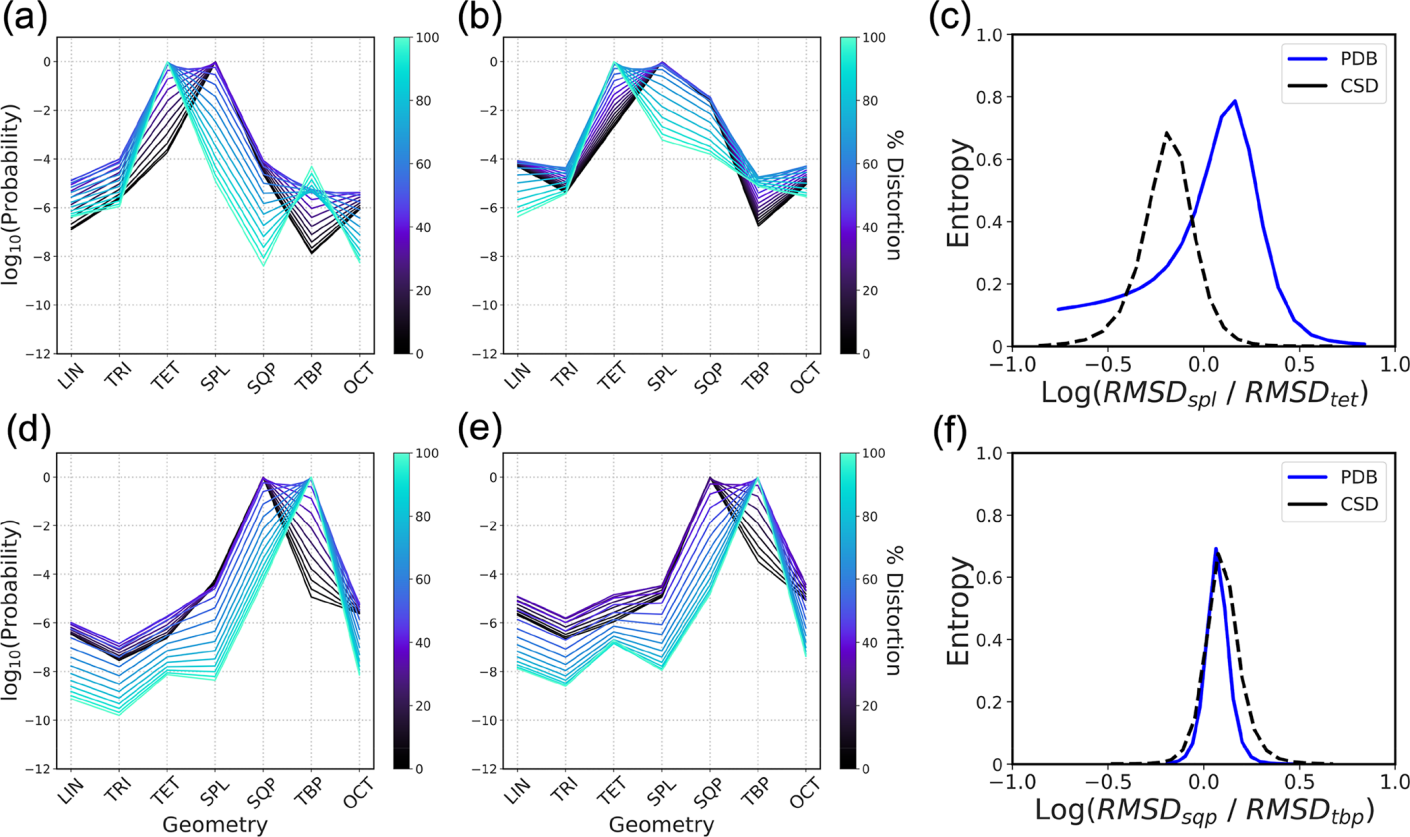
Evaluation of CSD-NN performance in response to SPL → TET (a-c) and SQP → TBP (d-f) metal site distortion. Evolution of class probability distributions outputted by CSD-NN with increasing metal site distortion for CSD entries AWOSUB01 (a) and CAVLAQ (d) and PDB entries 6R54 (b) and 6PMV (e). Entropy profiles for progressive distortion of SPL sites transformed into TET (c) and SQP sites transformed into TBP (f).

As an example of the effect of distortion on output probabilities and by extension on entropy, two pairs of SPL and SQP sites were randomly selected from the CSD and PDB validation sets to be distorted progressively into TET and TBP sites, respectively. The variation of probabilities output by CSD-NN with increasing distortion (Figure 6 a, b, d, and e) is perfectly consistent with the expected behavior. The initial probability distribution shows a clear maximum corresponding to the real class, progressively moving toward the class into which the site is being transformed.

Interestingly, inspection of the selected SPL PDB site (PDB ID: 6R54, chain A, residue number 303, atom name NI) revealed the presence of significant deviation from SPL ideal angles of the ligand atoms (162.4°, 176.5° instead of 180° and 98.2°, 97.4°, 82.5°, 82.4° instead of 90°), which is probably responsible for the somewhat skewed class-probability distribution assigned by the model. Entropy profiles as a function of distortion (Figure 6c, f), show a clear maximum located near RMSD ratio values corresponding to halfway between two geometries. Once again, the PDB SPL site shows higher entropy values, reflecting the higher uncertainty of the model in its class assignment, as discussed above.

### 3.4 Algorithm Benchmark and Comparison

In addition to evaluating algorithm performance, we also investigated the time necessary to perform metal site classification on both small metal complexes and metalloproteins.

**Figure 7:**
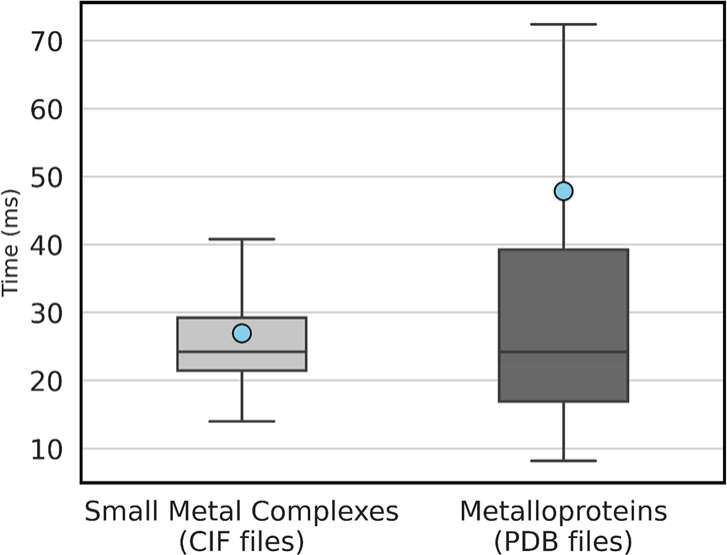
Total time required to classify metalloproteins (PDB files) and small metal complex (CIFs) using MetalHawk, from file parsing to classification of all metal sites in each file. Blue circles represent average values. Outliers are omitted for clarity. Classification times shown were obtained using a 6-core Intel Core i5 11400 processor.

The average total time required to classify all metal sites present in small complex and metalloprotein structures are 30 and 50 ms, respectively (Figure 7); however, some extremely large metalloprotein structures can require up to 2 s to complete classification.

The time required to perform classification alone, namely the time required by the input features to pass through the neural network, is very short, close to 0.25 ms on average, and independent of the CN and geometry of the metal site. Most of the time is therefore spent parsing the CIF or PDB file, extracting the coordinates of non-hydrogen atoms and determining the coordinates of the six nearest neighbors for each metal atom in the file. This last operation is performed through the KDTree neighbor lookup method,^68^ which is comparatively faster than other approaches, especially when a cutoff distance is specified (10 Å from the metal considered, in our implementation). Once the neighbors are found, input features can be easily calculated using Numpy and SciPy functions and assembled to form the 36-value input vector.

## 4 Conclusions

In this work, we show that ML-based models can be used to perform simultaneous geometry and coordination number assignment of both small complexes and metalloproteins. This represents the first example of such approach, to the best of our knowledge. We designed two simple ANNs, one trained on crystal structures deposited in the CSD (CSD-NN) and the other on metalloprotein structures from the PDB (PDB-NN). The CSD-NN model achieves near optimal classification accuracy on all considered classes, whereas the PDB-NN recognizes with high accuracy only the abundant TET and OCT classes, while being significantly less capable on less-represented geometries. Unlike PDB-NN, CSD-NN was shown to be a robust classifier for biomolecular sites in addition to metal complexes, with comparable accuracy on the two validation sets. Additionally, we have shown that entropy calculated from the probability distribution of the CSD-NN model can be interpreted as a measure of uncertainty pertaining to the predicted class, with higher entropy values correlated to a higher probability of misclassification and distorted coordination environments. The methods here described can in principle be extended to encompass higher CNs and more geometry classes, though generalization may be hampered by the scarcity of pertinent structural data. A number of strategies to compensate for class imbalance and insufficient support might also be attempted. These range from very simple approaches, such as including heavier elements into the CSD data set, to data augmentation solutions, such as replicating the metal sites from rare classes or generating artificial metal sites by introducing random distortions into the original data set entries to expand and artificially balance the different classes. These and other strategies will be attempted in the future, with the aim to develop a generalized model for fast and accurate annotation of metal site structural properties. Such a model would represent a powerful tool to facilitate the training of more complex ML-based models, ultimately aimed at *in silico* discovery of novel metal-based compounds for desirable applications.

## Data Availability

The weights of both the CSD-NN and PDB-NN models can be found at https://github.com/vrettasm/MetalHawk. All CSD and PDB metal sites used for training/validation and code to reproduce the figures shown in the main text are available in the Zenodo database at DOI: 10.5281/zenodo.8356896.

## Supporting Information Available

Geometrical description of the geometry classes considered, breakdown of composition and RMSD distribution in the training data; detailed performance of the models on the respective test sets and the optimized hyperparameter lists; *t* test scores for the entropy distributions calculated on the test sets; description of the procedure used to introduce distortions in metal sites and entropy variation as a function of structural distorsion; statistical correlations between entropy and RMSD/CShM; distribution of entropy values for correctly classified CSD validation set sites inside and outside the “twilight zone” (PDF)

## References

[ref1] Carbonell J. G.; Michalski R. S.; Mitchell T. M. AN OVERVIEW OF MACHINE LEARNING. In Machine Learning; Elsevier: 1983; pp 3–23, 10.1016/B978-0-08-051054-5.50005-4.

[ref2] Jordan M. I.; Mitchell T. M. Machine Learning: Trends, Perspectives, and Prospects. Science 2015, 349 (6245), 255–260. 10.1126/science.aaa8415. 26185243

[ref3] Libbrecht M. W.; Noble W. S. Machine Learning Applications in Genetics and Genomics. Nat. Rev. Genet. 2015, 16 (6), 321–332. 10.1038/nrg3920. 25948244 PMC5204302

[ref4] Tarca A. L.; Carey V. J.; Chen X.; Romero R.; Drăghici S. Machine Learning and Its Applications to Biology. PLoS Comput. Biol. 2007, 3 (6), e116 10.1371/journal.pcbi.0030116. 17604446 PMC1904382

[ref5] Vamathevan J.; Clark D.; Czodrowski P.; Dunham I.; Ferran E.; Lee G.; Li B.; Madabhushi A.; Shah P.; Spitzer M.; Zhao S. Applications of Machine Learning in Drug Discovery and Development. Nat. Rev. Drug Discovery 2019, 18 (6), 463–477. 10.1038/s41573-019-0024-5. 30976107 PMC6552674

[ref6] Shehab M.; Abualigah L.; Shambour Q.; Abu-Hashem M. A.; Shambour M. K. Y.; Alsalibi A. I.; Gandomi A. H. Machine Learning in Medical Applications: A Review of State-of-the-Art Methods. Comput. Biol. Med. 2022, 145, 105458 10.1016/j.compbiomed.2022.105458. 35364311

[ref7] Hassan-Harrirou H.; Zhang C.; Lemmin T. RosENet: Improving Binding Affinity Prediction by Leveraging Molecular Mechanics Energies with an Ensemble of 3D Convolutional Neural Networks. J. Chem. Inf. Model. 2020, 60 (6), 2791–2802. 10.1021/acs.jcim.0c00075. 32392050

[ref8] Nandy A.; Duan C.; Janet J. P.; Gugler S.; Kulik H. J. Strategies and Software for Machine Learning Accelerated Discovery in Transition Metal Chemistry. Ind. Eng. Chem. Res. 2018, 57 (42), 13973–13986. 10.1021/acs.iecr.8b04015.

[ref9] Janet J. P.; Kulik H. J. Resolving Transition Metal Chemical Space: Feature Selection for Machine Learning and Structure-Property Relationships. J. Phys. Chem. A 2017, 121 (46), 8939–8954. 10.1021/acs.jpca.7b08750. 29095620

[ref10] Kulik H. J. Making Machine Learning a Useful Tool in the Accelerated Discovery of Transition Metal Complexes. WIREs Comput. Mol. Sci. 2020, 10 (1), e1439 10.1002/wcms.1439.

[ref11] van Leeuwen P. W. N. M. Homogeneous Catalysis: Understanding the Art; Springer Netherlands: Dordrecht, 2004; 10.1007/1-4020-2000-7.

[ref12] James S. L. Metal-Organic Frameworks. Chem. Soc. Rev. 2003, 32 (5), 276–288. 10.1039/b200393g. 14518181

[ref13] Hagen J. Industrial Catalysis: A Practical Approach, 3rd completely revised and enlarged ed.; Wiley-VCH: Weinheim, 2015.

[ref14] Chorkendorff I.; Niemantsverdriet J. W. Concepts of Modern Catalysis and Kinetics, 1st ed.; Wiley: 2003; 10.1002/3527602658.

[ref15] Maglio O.; Nastri F.; Lombardi A. Structural and Functional Aspects of Metal Binding Sites in Natural and Designed Metalloproteins. In Ionic Interactions in Natural and Synthetic Macromolecules; Ciferri A., Perico A., Eds.; John Wiley & Sons, Inc.: Hoboken, NJ, USA, 2012; pp 361–450, 10.1002/9781118165850.ch11.

[ref16] Crichton R. R. Biological Inorganic Chemistry: A New Introduction to Molecular Structure and Function, Third ed.; Academic Press, an imprint of Elsevier: London, San Diego, CA, 2019.

[ref17] Cheng Y.; Wang H.; Xu H.; Liu Y.; Ma B.; Chen X.; Zeng X.; Wang X.; Wang B.; Shiau C.; Ovchinnikov S.; Su X.-D.; Wang C. Co-Evolution-Based Prediction of Metal-Binding Sites in Proteomes by Machine Learning. Nat. Chem. Biol. 2023, 19, 548–555. 10.1038/s41589-022-01223-z. 36593274

[ref18] Haberal I.; Ogul H. DeepMBS: Prediction of Protein Metal Binding-Site Using Deep Learning Networks. In 2017 Fourth International Conference on Mathematics and Computers in Sciences and in Industry (MCSI); IEEE: Corfu, 2017; pp 21–25, 10.1109/MCSI.2017.13.

[ref19] Haberal L̇.; Oğul H. Prediction of Protein Metal Binding Sites Using Deep Neural Networks. Mol. Inform. 2019, 38 (7), 1800169 10.1002/minf.201800169. 30977960

[ref20] Laveglia V.; Giachetti A.; Sala D.; Andreini C.; Rosato A. Learning to Identify Physiological and Adventitious Metal-Binding Sites in the Three-Dimensional Structures of Proteins by Following the Hints of a Deep Neural Network. J. Chem. Inf. Model. 2022, 62 (12), 2951–2960. 10.1021/acs.jcim.2c00522. 35679182 PMC9241070

[ref21] Koohi-Moghadam M.; Wang H.; Wang Y.; Yang X.; Li H.; Wang J.; Sun H. Predicting Disease-Associated Mutation of Metal-Binding Sites in Proteins Using a Deep Learning Approach. Nat. Mach. Intell. 2019, 1 (12), 561–567. 10.1038/s42256-019-0119-z.

[ref22] Wang J.; Lisanza S.; Juergens D.; Tischer D.; Watson J. L.; Castro K. M.; Ragotte R.; Saragovi A.; Milles L. F.; Baek M.; Anishchenko I.; Yang W.; Hicks D. R.; Expòsit M.; Schlichthaerle T.; Chun J.-H.; Dauparas J.; Bennett N.; Wicky B. I. M.; Muenks A.; DiMaio F.; Correia B.; Ovchinnikov S.; Baker D. Scaffolding Protein Functional Sites Using Deep Learning. Science 2022, 377 (6604), 387–394. 10.1126/science.abn2100. 35862514 PMC9621694

[ref23] Yu Y.; Wang R.; Teo R. D. Machine Learning Approaches for Metalloproteins. Molecules 2022, 27 (4), 1277 10.3390/molecules27041277. 35209064 PMC8878495

[ref24] Feehan R.; Franklin M. W.; Slusky J. S. G. Machine Learning Differentiates Enzymatic and Non-Enzymatic Metals in Proteins. Nat. Commun. 2021, 12 (1), 3712 10.1038/s41467-021-24070-3. 34140507 PMC8211803

[ref25] Mater A. C.; Coote M. L. Deep Learning in Chemistry. J. Chem. Inf. Model. 2019, 59 (6), 2545–2559. 10.1021/acs.jcim.9b00266. 31194543

[ref26] Carleo G.; Cirac I.; Cranmer K.; Daudet L.; Schuld M.; Tishby N.; Vogt-Maranto L.; Zdeborová L. Machine Learning and the Physical Sciences. Rev. Mod. Phys. 2019, 91 (4), 045002 10.1103/RevModPhys.91.045002.

[ref27] Alloghani M.; Al-Jumeily D.; Mustafina J.; Hussain A.; Aljaaf A. J. A Systematic Review on Supervised and Unsupervised Machine Learning Algorithms for Data Science. In Supervised and Unsupervised Learning for Data Science; Berry M. W., Mohamed A., Yap B. W., Eds.; Unsupervised and Semi-Supervised Learning; Springer International Publishing: Cham, 2020; pp 3–21, 10.1007/978-3-030-22475-2_1.

[ref28] Burley S. K.; Berman H. M.; Bhikadiya C.; Bi C.; Chen L.; Di Costanzo L.; Christie C.; Dalenberg K.; Duarte J. M.; Dutta S.; Feng Z.; Ghosh S.; Goodsell D. S.; Green R. K.; Guranović V.; Guzenko D.; Hudson B. P.; Kalro T.; Liang Y.; Lowe R.; Namkoong H.; Peisach E.; Periskova I.; Prlić A.; Randle C.; Rose A.; Rose P.; Sala R.; Sekharan M.; Shao C.; Tan L.; Tao Y.-P.; Valasatava Y.; Voigt M.; Westbrook J.; Woo J.; Yang H.; Young J.; Zhuravleva M.; Zardecki C. RCSB Protein Data Bank: Biological Macromolecular Structures Enabling Research and Education in Fundamental Biology, Biomedicine, Biotechnology and Energy. Nucleic Acids Res. 2019, 47 (D1), D464–D474. 10.1093/nar/gky1004. 30357411 PMC6324064

[ref29] Ward S. C.; Sadiq G. Introduction to the Cambridge Structural Database - a Wealth of Knowledge Gained from a Million Structures. CrystEngComm 2020, 22 (43), 7143–7144. 10.1039/D0CE90154G.

[ref30] Putignano V.; Rosato A.; Banci L.; Andreini C. MetalPDB in 2018: A Database of Metal Sites in Biological Macromolecular Structures. Nucleic Acids Res. 2018, 46 (D1), D459–D464. 10.1093/nar/gkx989. 29077942 PMC5753354

[ref31] Zimmermann N. E. R.; Horton M. K.; Jain A.; Haranczyk M. Assessing Local Structure Motifs Using Order Parameters for Motif Recognition, Interstitial Identification, and Diffusion Path Characterization. Front. Mater. 2017, 4, 34 10.3389/fmats.2017.00034.

[ref32] Brunner G. O. A Definition of Coordination and Its Relevance in the Structure Types AlB _2_ and NiAs. Acta Crystallogr., Sect. A 1977, 33 (1), 226–227. 10.1107/S0567739477000461.

[ref33] O’Keefe M.; Brese N. E. Atom Sizes and Bond Lengths in Molecules and Crystals. J. Am. Chem. Soc. 1991, 113 (9), 3226–3229. 10.1021/ja00009a002.

[ref34] Pan H.; Ganose A. M.; Horton M.; Aykol M.; Persson K. A.; Zimmermann N. E. R.; Jain A. Benchmarking Coordination Number Prediction Algorithms on Inorganic Crystal Structures. Inorg. Chem. 2021, 60 (3), 1590–1603. 10.1021/acs.inorgchem.0c02996. 33417450

[ref35] O’Keeffe M. A Proposed Rigorous Definition of Coordination Number. Acta Crystallogr. A 1979, 35 (5), 772–775. 10.1107/S0567739479001765.

[ref36] Zabrodsky H.; Peleg S.; Avnir D. Continuous Symmetry Measures. J. Am. Chem. Soc. 1992, 114 (20), 7843–7851. 10.1021/ja00046a033.

[ref37] Alvarez S.; Alemany P.; Casanova D.; Cirera J.; Llunell M.; Avnir D. Shape Maps and Polyhedral Interconversion Paths in Transition Metal Chemistry. Coord. Chem. Rev. 2005, 249 (17–18), 1693–1708. 10.1016/j.ccr.2005.03.031.

[ref38] Casanova D.; Cirera J.; Llunell M.; Alemany P.; Avnir D.; Alvarez S. Minimal Distortion Pathways in Polyhedral Rearrangements. J. Am. Chem. Soc. 2004, 126 (6), 1755–1763. 10.1021/ja036479n. 14871107

[ref39] Andreini C.; Cavallaro G.; Lorenzini S. FindGeo: A Tool for Determining Metal Coordination Geometry. Bioinformatics 2012, 28 (12), 1658–1660. 10.1093/bioinformatics/bts246. 22556364

[ref40] Zheng H.; Cooper D. R.; Porebski P. J.; Shabalin I. G.; Handing K. B.; Minor W. CheckMyMetal: A Macromolecular Metal-Binding Validation Tool. Acta Crystallogr. Sect. Struct. Biol. 2017, 73 (3), 223–233. 10.1107/S2059798317001061. PMC5349434 28291757

[ref41] Waroquiers D.; George J.; Horton M.; Schenk S.; Persson K. A.; Rignanese G.-M.; Gonze X.; Hautier G. *ChemEnv* : A Fast and Robust Coordination Environment Identification Tool. Acta Crystallogr. Sect. B Struct. Sci. Cryst. Eng. Mater. 2020, 76 (4), 683–695. 10.1107/S2052520620007994. PMC7412753 32831287

[ref42] Ong S. P.; Richards W. D.; Jain A.; Hautier G.; Kocher M.; Cholia S.; Gunter D.; Chevrier V. L.; Persson K. A.; Ceder G. Python Materials Genomics (Pymatgen): A Robust, Open-Source Python Library for Materials Analysis. Comput. Mater. Sci. 2013, 68, 314–319. 10.1016/j.commatsci.2012.10.028.

[ref43] Meng G.; Lam N. Y. S.; Lucas E. L.; Saint-Denis T. G.; Verma P.; Chekshin N.; Yu J.-Q. Achieving Site-Selectivity for C-H Activation Processes Based on Distance and Geometry: A Carpenter’s Approach. J. Am. Chem. Soc. 2020, 142 (24), 10571–10591. 10.1021/jacs.0c04074. 32437604 PMC7485751

[ref44] Nandy A.; Adamji H.; Kastner D. W.; Vennelakanti V.; Nazemi A.; Liu M.; Kulik H. J. Using Computational Chemistry To Reveal Nature’s Blueprints for Single-Site Catalysis of C-H Activation. ACS Catal. 2022, 12 (15), 9281–9306. 10.1021/acscatal.2c02096.

[ref45] Chalkley M. J.; Mann S. I.; DeGrado W. F. De Novo Metalloprotein Design. Nat. Rev. Chem. 2022, 6 (1), 31–50. 10.1038/s41570-021-00339-5. 35811759 PMC9264687

[ref46] Koebke K. J.; Pinter T. B. J.; Pitts W. C.; Pecoraro V. L. Catalysis and Electron Transfer in *De Novo* Designed Metalloproteins. Chem. Rev. 2022, 122 (14), 12046–12109. 10.1021/acs.chemrev.1c01025. 35763791 PMC10735231

[ref47] Nastri F.; D’Alonzo D.; Leone L.; Zambrano G.; Pavone V.; Lombardi A. Engineering Metalloprotein Functions in Designed and Native Scaffolds. Trends Biochem. Sci. 2019, 44 (12), 1022–1040. 10.1016/j.tibs.2019.06.006. 31307903

[ref48] Lombardi A.; Pirro F.; Maglio O.; Chino M.; DeGrado W. F. De Novo Design of Four-Helix Bundle Metalloproteins: One Scaffold, Diverse Reactivities. Acc. Chem. Res. 2019, 52 (5), 1148–1159. 10.1021/acs.accounts.8b00674. 30973707 PMC7362765

[ref49] Chino M.; Di Costanzo L. F.; Leone L.; La Gatta S.; Famulari A.; Chiesa M.; Lombardi A.; Pavone V. Designed Rubredoxin Miniature in a Fully Artificial Electron Chain Triggered by Visible Light. Nat. Commun. 2023, 14 (1), 2368 10.1038/s41467-023-37941-8. 37185349 PMC10130062

[ref50] Pirro F.; La Gatta S.; Arrigoni F.; Famulari A.; Maglio O.; Del Vecchio P.; Chiesa M.; De Gioia L.; Bertini L.; Chino M.; Nastri F.; Lombardi A. A De Novo Designed Type 3 Copper Protein Tunes Catechol Substrate Recognition and Reactivity. Angew. Chem. 2023, 135 (1), e202211552 10.1002/ange.202211552. 36334012

[ref51] Krogh A. What Are Artificial Neural Networks?. Nat. Biotechnol. 2008, 26 (2), 195–197. 10.1038/nbt1386. 18259176

[ref52] Groom C. R.; Bruno I. J.; Lightfoot M. P.; Ward S. C. The Cambridge Structural Database. Acta Crystallogr. Sect. B Struct. Sci. Cryst. Eng. Mater. 2016, 72 (2), 171–179. 10.1107/S2052520616003954. PMC4822653 27048719

[ref53] Berman H. M. The Protein Data Bank. Nucleic Acids Res. 2000, 28 (1), 235–242. 10.1093/nar/28.1.235. 10592235 PMC102472

[ref54] Bruno I. J.; Cole J. C.; Edgington P. R.; Kessler M.; Macrae C. F.; McCabe P.; Pearson J.; Taylor R. New Software for Searching the Cambridge Structural Database and Visualizing Crystal Structures. Acta Crystallogr. B 2002, 58 (3), 389–397. 10.1107/S0108768102003324. 12037360

[ref55] Andreini C.; Cavallaro G.; Lorenzini S.; Rosato A. MetalPDB: A Database of Metal Sites in Biological Macromolecular Structures. Nucleic Acids Res. 2012, 41 (D1), D312–D319. 10.1093/nar/gks1063. 23155064 PMC3531106

[ref56] Pedregosa F.; Varoquaux G.; Gramfort A.; Michel V.; Thirion B.; Grisel O.; Blondel M.; Prettenhofer P.; Weiss R.; Dubourg V.; Vanderplas J.; Passos A.; Cournapeau D.; Brucher M.; Perrot M.; Duchesnay É. Scikit-Learn: Machine Learning in Python. J. Mach. Learn. Res. 2011, 12 (85), 2825–2830.

[ref57] Shannon C. E. A Mathematical Theory of Communication. Bell Syst. Technol. J. 1948, 27 (4), 623–656. 10.1002/j.1538-7305.1948.tb00917.x.

[ref58] Harris C. R.; Millman K. J.; van der Walt S. J.; Gommers R.; Virtanen P.; Cournapeau D.; Wieser E.; Taylor J.; Berg S.; Smith N. J.; Kern R.; Picus M.; Hoyer S.; van Kerkwijk M. H.; Brett M.; Haldane A.; del Río J. F.; Wiebe M.; Peterson P.; Gérard-Marchant P.; Sheppard K.; Reddy T.; Weckesser W.; Abbasi H.; Gohlke C.; Oliphant T. E. Array Programming with NumPy. Nature 2020, 585 (7825), 357–362. 10.1038/s41586-020-2649-2. 32939066 PMC7759461

[ref59] Alemany P.; Bernuz E.; Carreras A.; Llunell M. Cosymlib: A Python Library for Continuous Symmetry Measures; 2021; 10.5281/ZENODO.4925767.

[ref60] Shabalin I.; Dauter Z.; Jaskolski M.; Minor W.; Wlodawer A. Crystallography and Chemistry Should Always Go Together: A Cautionary Tale of Protein Complexes with Cisplatin and Carboplatin. Acta Crystallogr. D Biol. Crystallogr. 2015, 71 (9), 1965–1979. 10.1107/S139900471500629X. 26327386 PMC4556316

[ref61] Wlodawer A.; Dauter Z.; Porebski P. J.; Minor W.; Stanfield R.; Jaskolski M.; Pozharski E.; Weichenberger C. X.; Rupp B. Detect, Correct, Retract: How to Manage Incorrect Structural Models. FEBS J. 2018, 285 (3), 444–466. 10.1111/febs.14320. 29113027 PMC5799025

[ref62] Bowman S. E. J.; Bridwell-Rabb J.; Drennan C. L. Metalloprotein Crystallography: More than a Structure. Acc. Chem. Res. 2016, 49 (4), 695–702. 10.1021/acs.accounts.5b00538. 26975689 PMC4838947

[ref63] Rulıšek L.; Vondrášek J. Coordination Geometries of Selected Transition Metal Ions (Co2+, Ni2+, Cu2+, Zn2+, Cd2+, and Hg2+) in Metalloproteins. J. Inorg. Biochem. 1998, 71 (3–4), 115–127. 10.1016/S0162-0134(98)10042-9. 9833317

[ref64] Harding M. M. Small Revisions to Predicted Distances around Metal Sites in Proteins. Acta Crystallogr. D Biol. Crystallogr. 2006, 62 (6), 678–682. 10.1107/S0907444906014594. 16699196

[ref65] Zheng H.; Chruszcz M.; Lasota P.; Lebioda L.; Minor W. Data Mining of Metal Ion Environments Present in Protein Structures. J. Inorg. Biochem. 2008, 102 (9), 1765–1776. 10.1016/j.jinorgbio.2008.05.006. 18614239 PMC2872550

[ref66] Hsin K.; Sheng Y.; Harding M. M.; Taylor P.; Walkinshaw M. D. MESPEUS: A Database of the Geometry of Metal Sites in Proteins. J. Appl. Crystallogr. 2008, 41 (5), 963–968. 10.1107/S002188980802476X.

[ref67] Harding M. M. Geometry of Metal-Ligand Interactions in Proteins. Acta Crystallogr. D Biol. Crystallogr. 2001, 57 (3), 401–411. 10.1107/S0907444900019168. 11223517

[ref68] Maneewongvatana S.; Mount D. M. Analysis of Approximate Nearest Neighbor Searching with Clustered Point Sets. arXiv:cs/9901013. 1999. ArXiv preprint. https://arxiv.org/abs/cs/9901013 (accessed 2023-11-09).

