# Supplementary material for: MetalHawk: Enhanced Classification of Metal Coordination Geometries by Artificial Neural Networks
Source: J Chem Inf Model. 2023 Nov 13;64(7):2356–67. doi: 10.1021/acs.jcim.3c00873 (PMC11005052; doi:10.1021/acs.jcim.3c00873)
Supplement: Supplementary file 1 — ci3c00873_si_001.pdf [file ci3c00873_si_001.pdf]

# Supporting information

## MetalHawk: Enhanced Classification Of Metal Coordination Geometries by Artificial Neural Networks

Gianmattia Sgueglia[†,§], Michail D. Vrettas[‡,§], Marco Chino[†], Alfonso De Simone[‡,1] and Angela Lombardi[†,2]

*† Department of Chemical Sciences, University of Naples Federico II, Via Cintia 21, 80126 Napoli, Italy.*

*‡ Department of Pharmacy, University of Naples Federico II, Via Domenico Montesano 49, 80131 Napoli, Italy.*

Corresponding Authors Email: alfonso.desimone@unina.it[1], alombard@unina.it[2]

## SI 1. Artificial distortion of existing metal sites

Metal sites from the CSD and PDB validation sets were distorted by manipulating the cartesian coordinates of specific ligand atoms, progressively morphing the original coordination geometry into the desired one. The following passage only describes the details of the distortion trajectory to transform SPL sites in TET sites and SQP sites in TBP sites, since the inverse transformations can be simply envisioned by reversing the procedures outlined.

SPL sites were aligned to the reference system of Cartesian axes placing the four ligand atoms (Figure S1 (a)) along the y and z directions and the metal atom on the origin. Ligands placed along the y axis (L2 and L4) were rotated around the z axis in opposite directions compared to each other, thereby compressing the L2-M-L4 angle. Ligands placed instead along the z axis (L1 and L3) were rotated around the y axis to compress the L1-M-L3 angle. The rotation step was determined by subtracting the desired value (109.5° for a perfect tetrahedron) from the initial angle formed by the two pairs of ligand atoms and dividing the resulting angle by 20.

A similar procedure was followed to distort the SQP sites (Figure S1 (b)), but only rotating ligands placed along the y axis (L2 and L4) around the z axis. This resulted in the compression of the L2-M-L4, which was gradually brought as close as possible to the desired value (120° for equatorial ligands in a perfect trigonal bipyramid) by stepwise rotation for 20 steps. The coordinates of the six metal atom nearest neighbors were recorded for all four sites at each step to calculate the input vectors, which were subsequently passed to CSD-NN to compute the probability assigned for each class and the entropy of the output vectors. All calculations necessary for the distortions were performed with a custom python script using Scipy, Numpy, pymatgen and cosymlib. The procedure to transform metal site geometry described above was applied to 213

TET, 183 SPL, 89 SQP and 132 TBP sites from the CSD validation set and to 20 TET, 9 SPL, 10 SQP and 14 TBP sites from the PDB validation set. Entropy was calculated for the original and all distorted structures and represented as heatmaps (Figure S2) and as boxplots divided into bins of CShMs ratios (Figure S3). Finally, wo pairs of SPL and SQP sites (Figure S4) were randomly chosen from the CSD and PDB validation sets to also show detailed class probability profiles and entropy evolution during the progress of the distortion trajectory (Main Text, Figure 6).

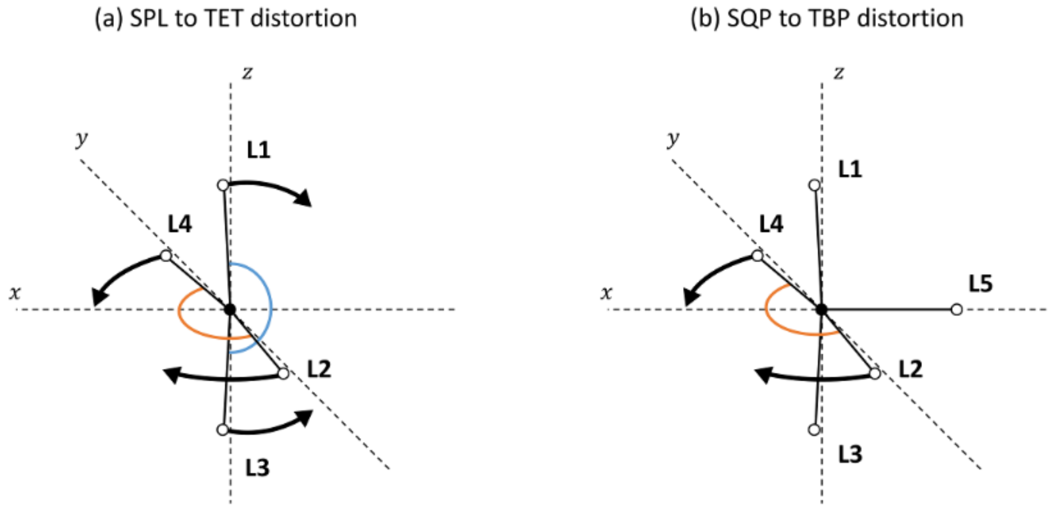


**Figure S1**: Distortion of SPL sites to TET and of SQP sites to TBP. Colored arcs represent angles subject to variation during the distortion trajectory.

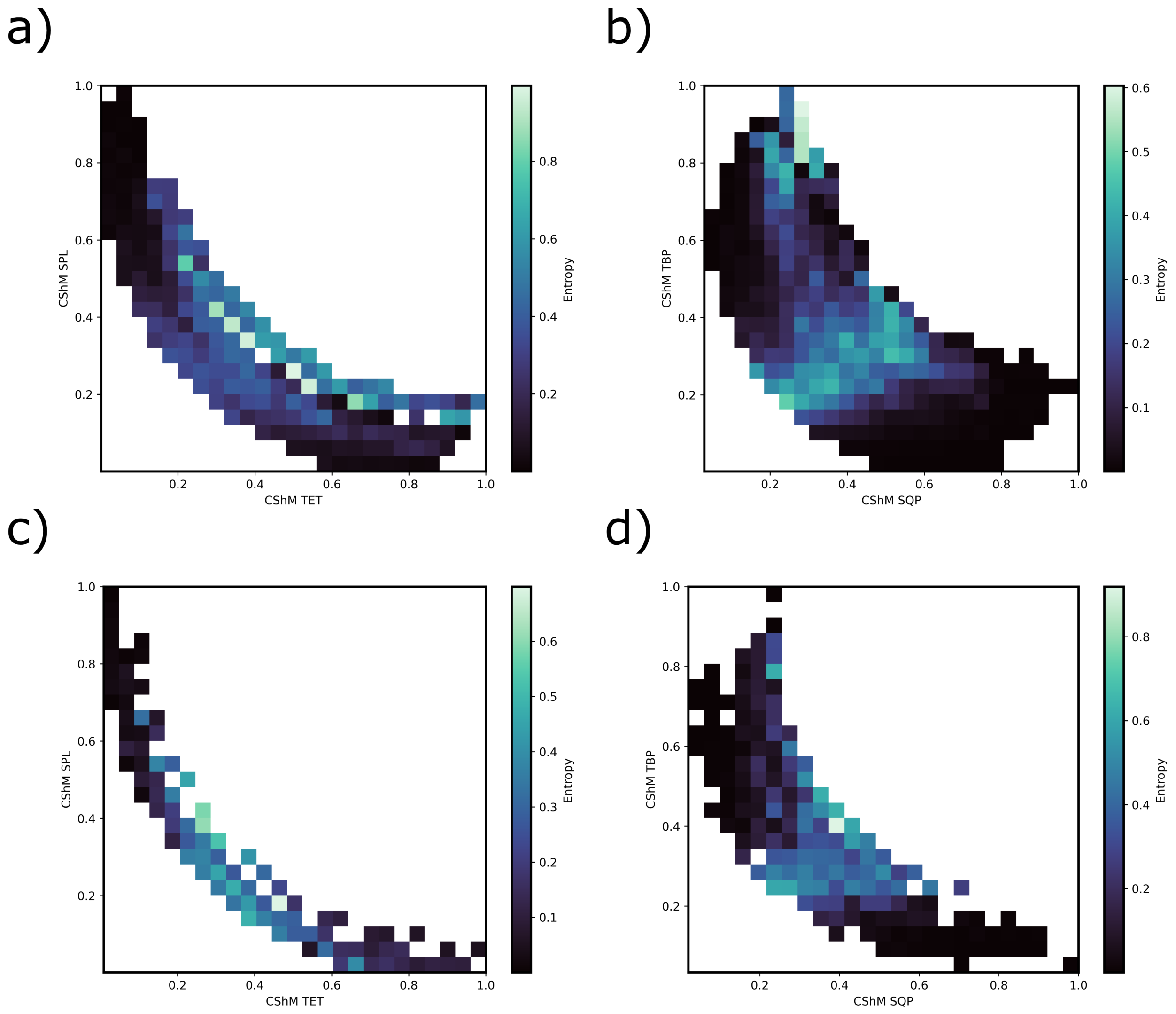


**Figure S2**: Entropy heatmaps for TET↔SPL distortion trajectories for a) CSD and c) PDB validation set sites and SQP↔TBP distortion trajectories for b) CSD and d) PDB validation set sites

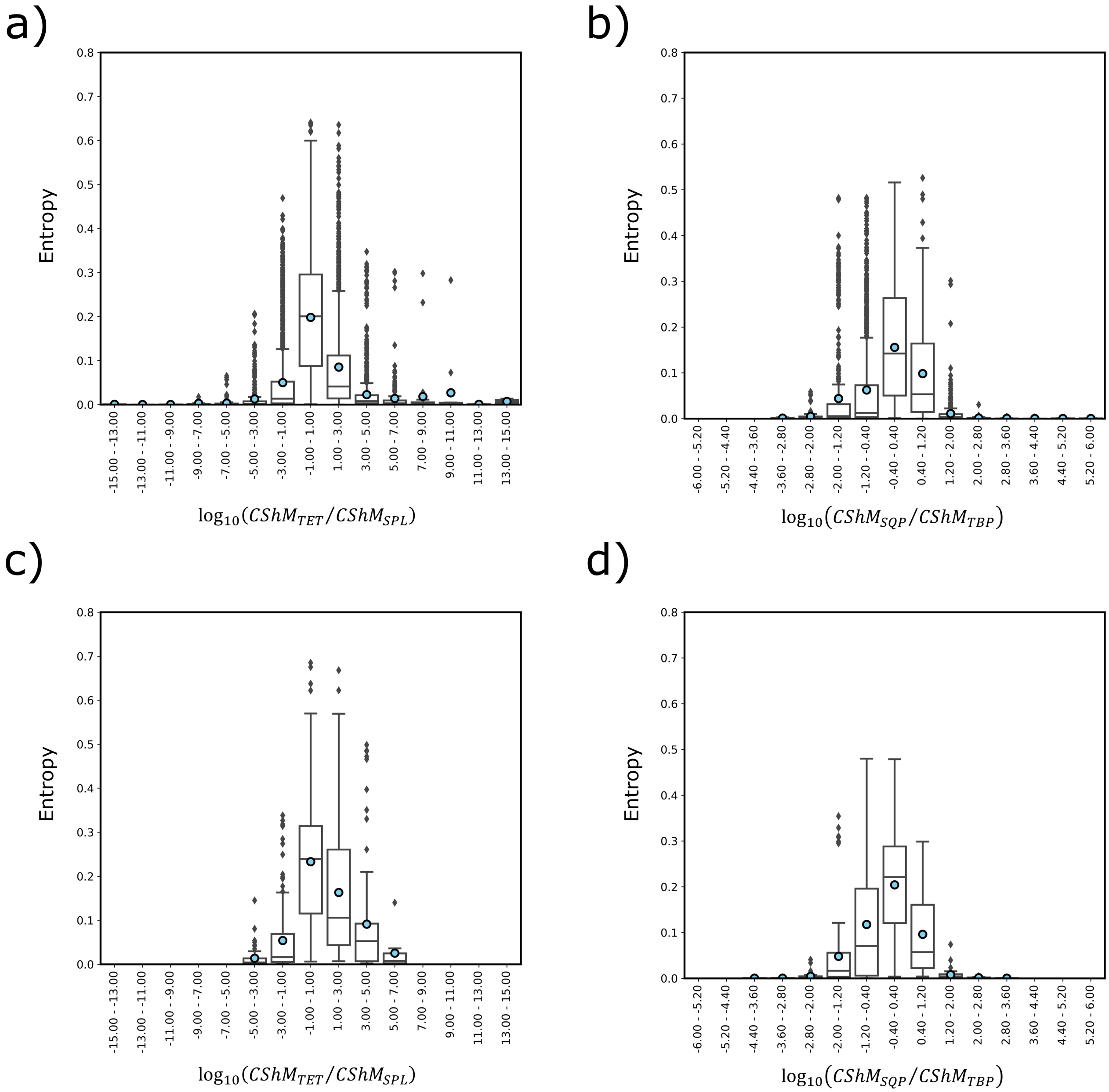


**Figure S3**: Entropy distribution boxplots for TET↔SPL distortion trajectories for a) CSD and c) PDB validation set sites and SQP↔TBP distortion trajectories for b) CSD and d) PDB validation set sites. Blue circles represent average values for each bin while black diamonds represent outliers.

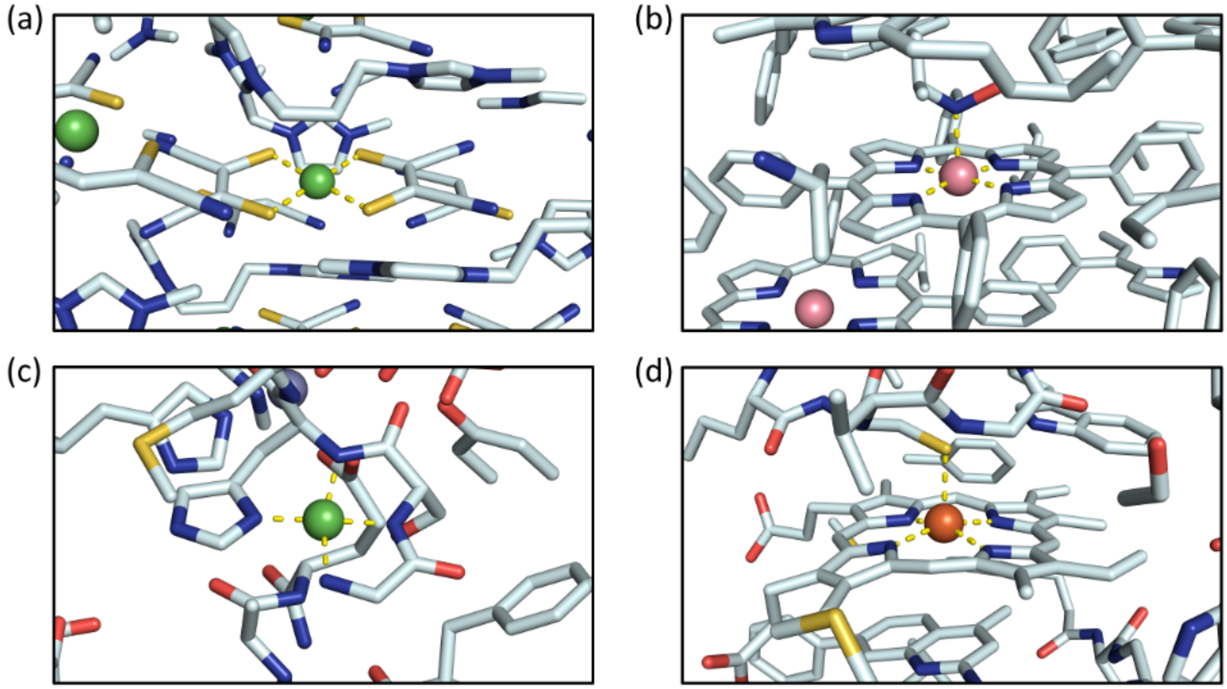


**Figure S4**: Metal sites on which distortions were performed to study the relationship between structural distortion and entropy. (a) CSD nickel square planar site (ID: AWOSUB01) , (b) CSD square pyramidal cobalt site (ID: CAVLAQ), (c) PDB nickel square planar site (ID: 6R54) and (d) PDB iron square pyramidal site (ID: 6PMV). All metal sites shown were randomly chosen from the CSD and PDB validation sets.

**Table S1**: List of coordination geometries considered in this work, their coordination numbers and the ideal angles formed by pairs of ligand atoms and having the metal atom as vertex (L-M-L angles).

| Geometry class | Coordination Number | L-M-L angles |
|---|---|---|
| Linear (LIN)<br>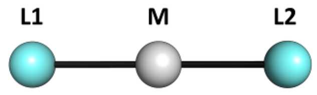<br> | 2 | L1-M-L2= **180°** |
| Trigonal planar (TRI)<br>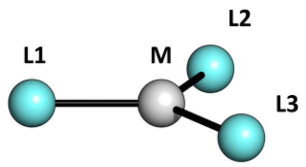<br> | 3 | L1-M-L2=L2-M-L3=<br>L1-M-L3= **120°** |

| | | |
|---|---|---|
| Tetrahedral (TET)<br>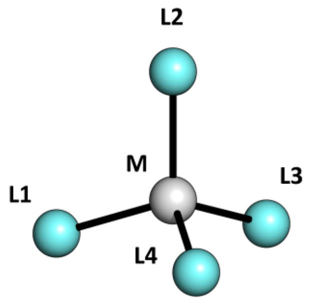 | 4 | L1-M-L2=L2-M-L3=<br>L3-M-L4=L4-M-L1=<br>L1-M-L3=L2-M-L4=<br>**109.5°** |
| Square planar (SPL)<br>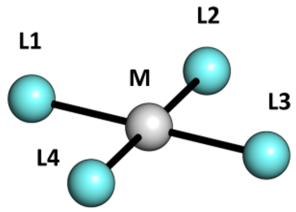 | 4 | L1-M-L2=L2-M-L3=<br>L3-M-L4=L4-M-L1=<br>**90°**<br>L1-M-L3=L2-M-L4=<br>**180°** |
| Square pyramidal (SQP)<br>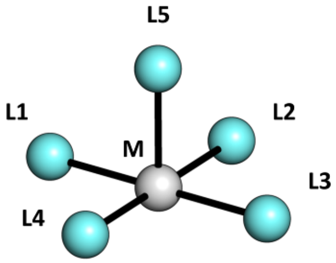 | 5 | L1-M-L2=L2-M-L3=<br>L3-M-L4=L4-M-L1=<br>**90°**<br>L1-M-L3=L2-M-L4=<br>**180°**<br>L1-M-L5=L2-M-L5=<br>L3-M-L5=L4-M-L5=<br>**90°** |

| | | |
|---|---|---|
| Trigonal bipyramidal (TBP) 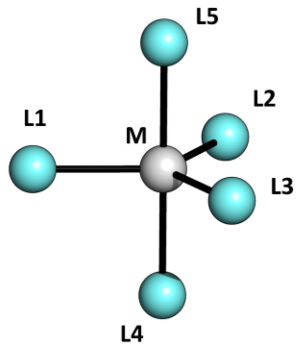  | 5 | L1-M-L2=L2-M-L3= L1-M-L3= **120°**<br>L1-M-L4=L2-M-L4= L3-M-L4= **90°**<br>L1-M-L5=L2-M-L5= L3-M-L5= **90°**<br>L4-M-L5= **180°** |
| Octahedral (OCT) 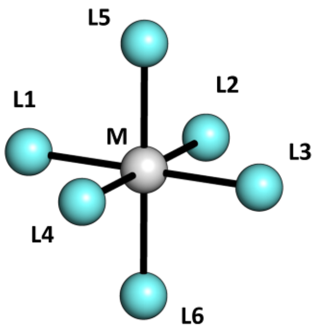  | 6 | L1-M-L2=L2-M-L3= L3-M-L4=L4-M-L1= **90°**<br>L1-M-L3=L2-M-L4= L5-M-L6= **180°**<br>L1-M-L5=L2-M-L5= L3-M-L5=L4-M-L5= **90°**<br>L1-M-L6=L2-M-L6= L3-M-L6=L4-M-L6= **90°** |

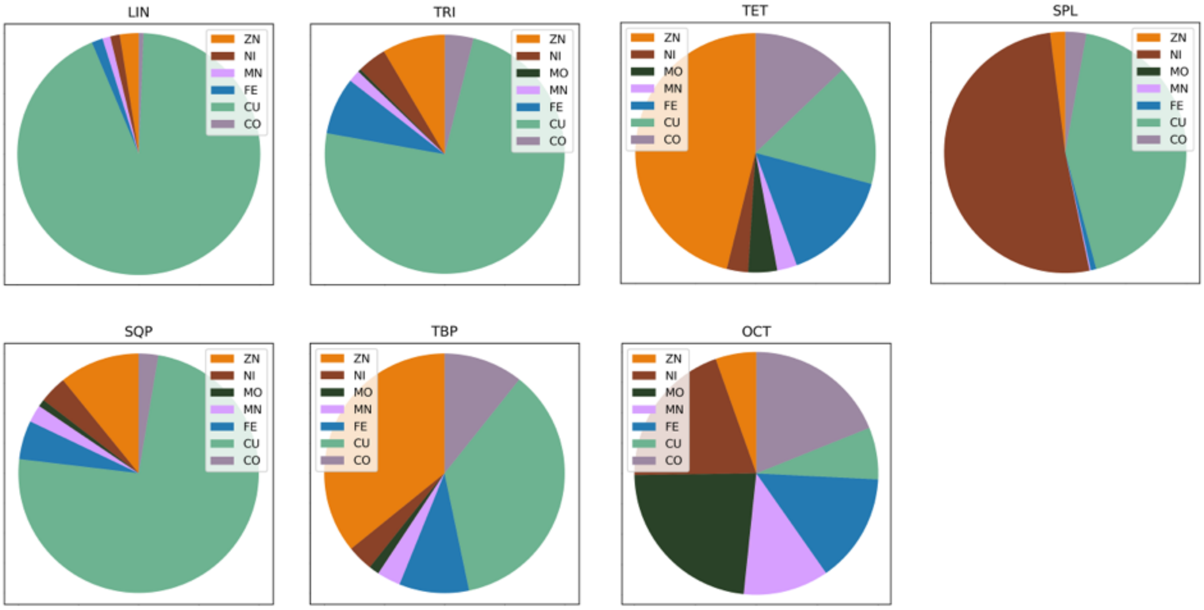


**Figure S5:** Per class distribution of metal identity across the entire CSD dataset.

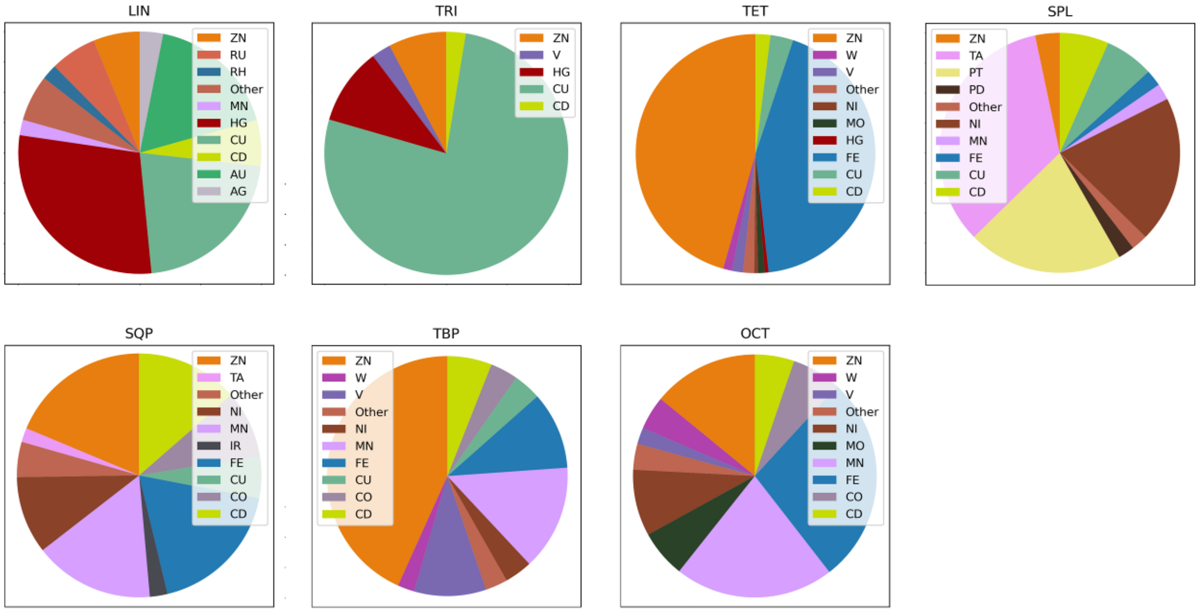


**Figure S6:** Per class distribution of metal identity across the entire PDB dataset.

(a)

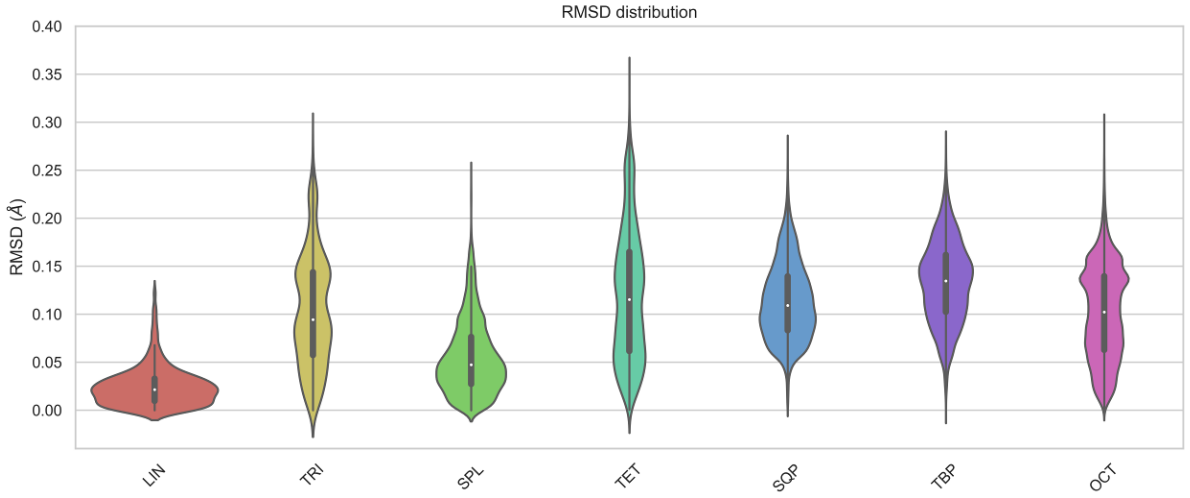


(b)

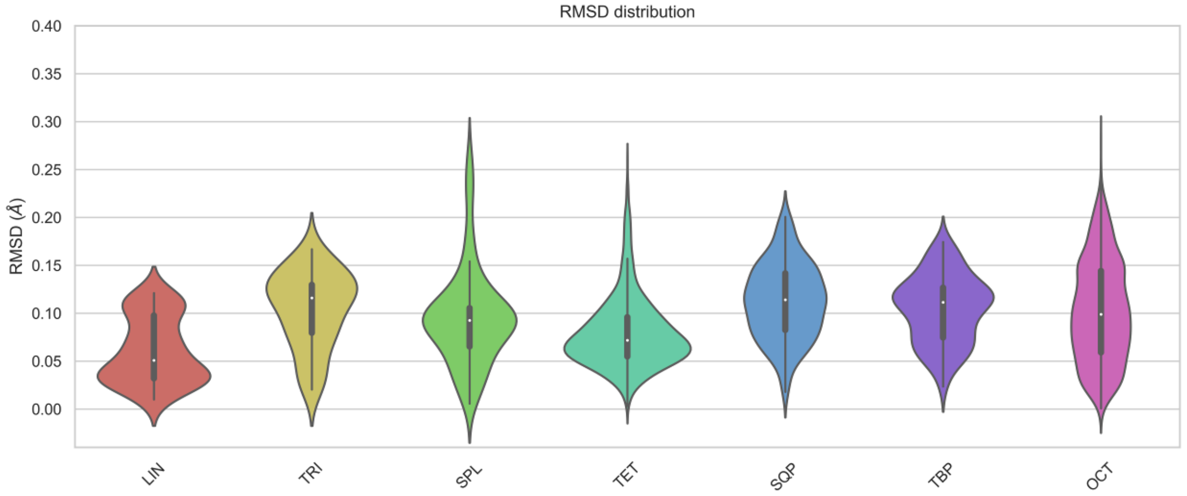


**Figure S7**: Per class distribution of RMSD computed against the idealized class models for preliminary classification of the CSD dataset (a) and PDB dataset (b). RMSD values were calculated using the BFOM algorithm available in the Pymatgen package, after scaling the length of all metal-ligand bonds to 2 Å.

**Table S2**: Balanced accuracy scores of optimized models computed on the full training sets for a) CSD sites and b) PDB sites in cross-validation. Standard deviation is shown in parenthesis next to mean value.

a)

| | |
|---|---|
| Fold 1 | 0.867860530103233 |
| Fold 2 | 0.888578041169576 |
| Fold 3 | 0.833888350873674 |
| Fold 4 | 0.927136067415899 |
| Fold 5 | 0.895447344364044 |
| Mean | 0.8826 (0.0309) |

b)

| | |
|---|---|
| Fold 1 | 0.9821713894047751 |
| Fold 2 | 0.9841607732275625 |
| Fold 3 | 0.9842307976888210 |
| Fold 4 | 0.9860724585476197 |
| Fold 5 | 0.9792345648299617 |
| Mean | 0.9832 (0.0023) |

**Table S3**: List of parameters for optimized CSD-NN implemented in scikit-learn.

| | |
|---|---|
| 'data_scaler' | RobustScaler() |
| 'mlp' | MLPClassifier() |
| 'data_scaler__copy' | True |
| 'data_scaler__quantile_range' | (25.0 75.0) |
| 'data_scaler__unit_variance' | False |
| 'data_scaler__with_centering' | True |
| 'data_scaler__with_scaling' | True |
| 'mlp__activation' | 'tanh' |
| 'mlp__alpha' | 0.1 |
| 'mlp__batch_size' | 1024 |
| 'mlp__beta_1' | 0.9 |
| 'mlp__beta_2' | 0.999 |
| 'mlp__early_stopping' | False |
| 'mlp__epsilon' | 1e-08 |
| 'mlp__hidden_layer_sizes' | 1024 |
| 'mlp__learning_rate' | 'constant' |
| 'mlp__learning_rate_init' | 0.0013556613475402466 |
| 'mlp__max_fun' | 15000 |
| 'mlp__max_iter' | 200 |
| 'mlp__momentum' | 0.9 |
| 'mlp__n_iter_no_change' | 10 |
| 'mlp__nesterovs_momentum' | True |
| 'mlp__power_t' | 0.5 |
| 'mlp__random_state' | None |
| 'mlp__shuffle' | True |
| 'mlp__solver' | 'adam' |
| 'mlp__tol' | 0.0001 |
| 'mlp__validation_fraction' | 0.1 |
| 'mlp__verbose' | False |
| 'mlp__warm_start' | False |

**Table S4**: List of parameters for optimized PDB-NN implemented in scikit-learn.

| | |
|---|---|
| 'data_scaler' | RobustScaler() |
| 'mlp' | MLPClassifier() |
| 'data_scaler__copy' | True |
| 'data_scaler__quantile_range' | (25.0 75.0) |
| 'data_scaler__unit_variance' | False |
| 'data_scaler__with_centering' | True |
| 'data_scaler__with_scaling' | True |
| 'mlp__activation' | 'tanh' |
| 'mlp__alpha' | 0.0001 |
| 'mlp__batch_size' | 762 |
| 'mlp__beta_1' | 0.9 |
| 'mlp__beta_2' | 0.999 |
| 'mlp__early_stopping' | False |
| 'mlp__epsilon' | 1e-08 |
| 'mlp__hidden_layer_sizes' | 1024 |
| 'mlp__learning_rate' | 'constant' |
| 'mlp__learning_rate_init' | 0.001 |
| 'mlp__max_fun' | 15000 |
| 'mlp__max_iter' | 200 |
| 'mlp__momentum' | 0.9 |
| 'mlp__n_iter_no_change' | 10 |
| 'mlp__nesterovs_momentum' | True |
| 'mlp__power_t' | 0.5 |
| 'mlp__random_state' | None |
| 'mlp__shuffle' | True |
| 'mlp__solver' | 'adam' |
| 'mlp__tol' | 0.0001 |
| 'mlp__validation_fraction' | 0.1 |
| 'mlp__verbose' | False |
| 'mlp__warm_start' | False |

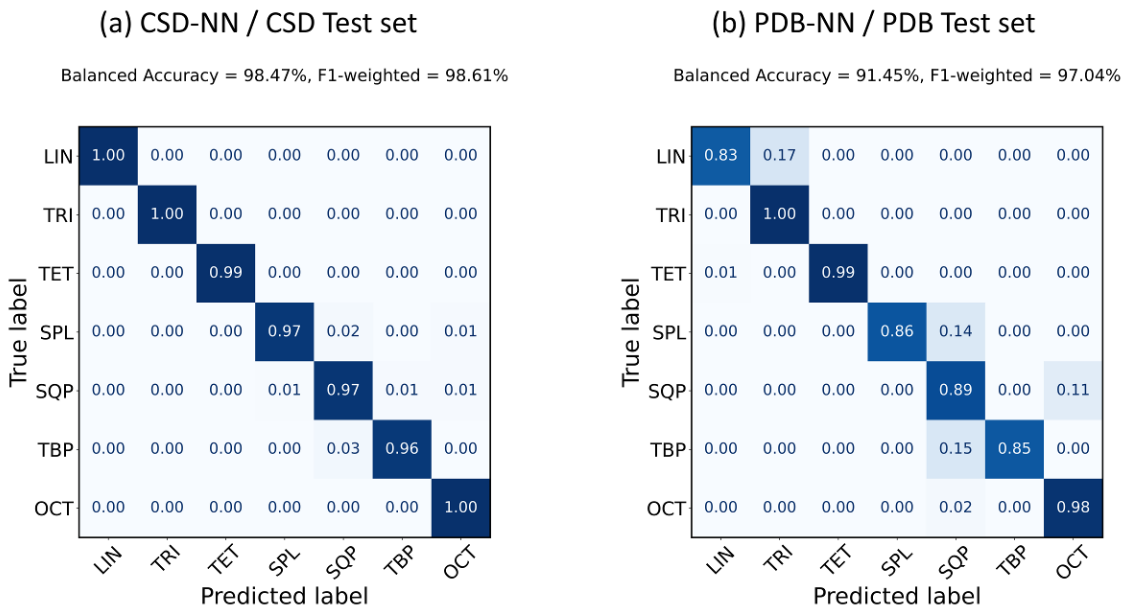


**Figure S8**: Full confusion matrices for classification by the CSD-NN model on the CSD test set (a) and for the PDB-NN model on the PDB test set.

**Table S5**: p-values of the t-test performed on the entropy values calculated by the CSD-NN model on the CSD test set and by the PDB-NN model on the PDB test set for each geometry class.

| Class | p-value |
|---|---|
| LIN | 0.5422250838215643 |
| TRI | 0.0005641844829392631 |
| TET | 8.492568769644028e-07 |
| SPL | 4.770863387557216e-09 |
| SQP | 4.029010291634071e-08 |
| TBP | 8.523279421282951e-07 |
| OCT | 1.3470862338247164e-41 |

**Table S6**: Performance metrics on the whole PDB dataset for hyper-parameter optimized CSD-NN model and support for each class.

| | Precision | Recall | F1-score | Support |
|---|---|---|---|---|
| LIN | 0.9255 | 0.8969 | 0.9110 | 97 |
| TRI | 0.6250 | 0.8974 | 0.7368 | 39 |
| TET | 0.9858 | 0.9841 | 0.9850 | 1198 |
| SPL | 0.8776 | 0.4725 | 0.6143 | 91 |
| SQP | 0.6982 | 0.7243 | 0.7110 | 214 |
| TBP | 0.8323 | 0.9627 | 0.8927 | 134 |
| OCT | 0.9613 | 0.9621 | 0.9617 | 1187 |
| Macro Average | 0.8437 | 0.8429 | 0.8304 | |
| Weighted Average | 0.9382 | 0.9358 | 0.9346 | 2960 |

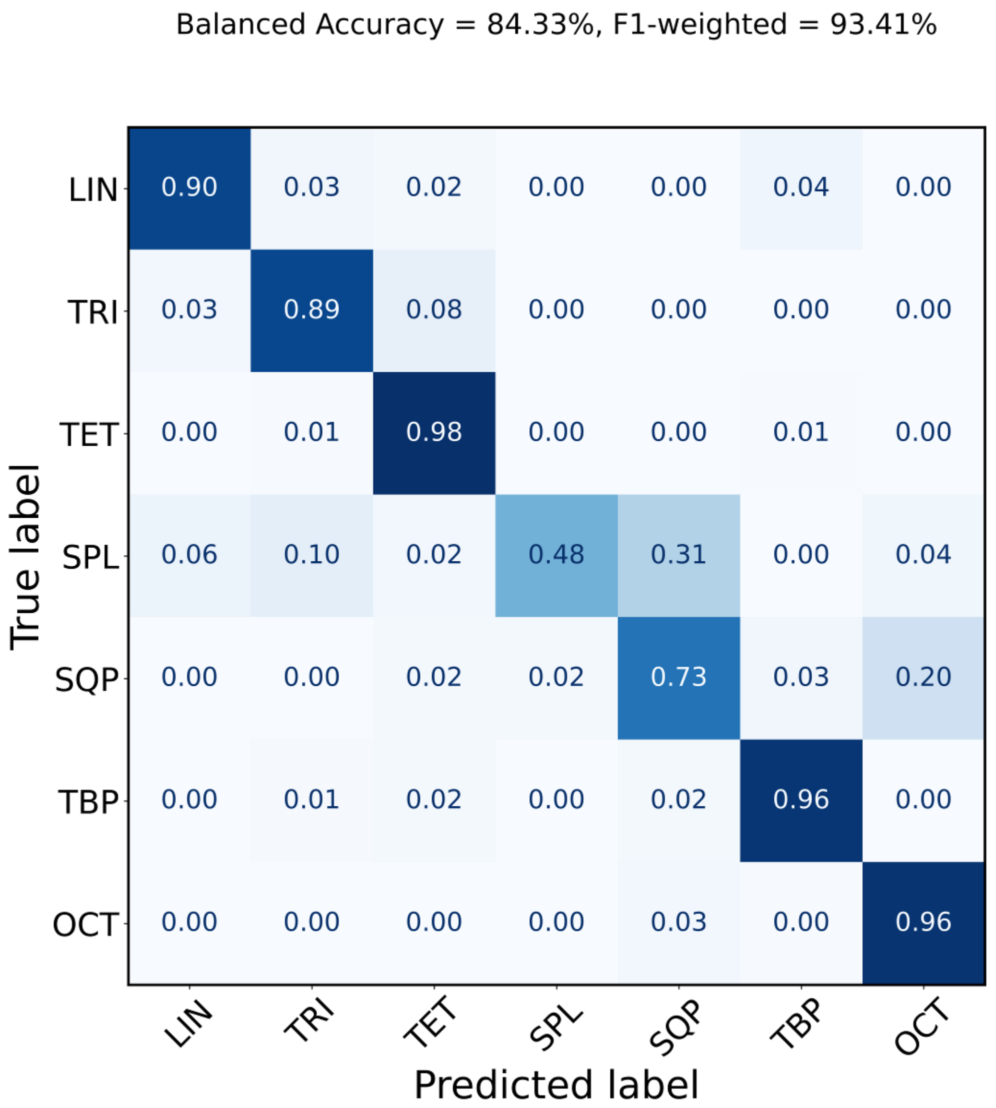


**Figure S9**: Full confusion matrices for classification by the CSD-NN model on the whole PDB dataset (PDB training set + PDB test set).

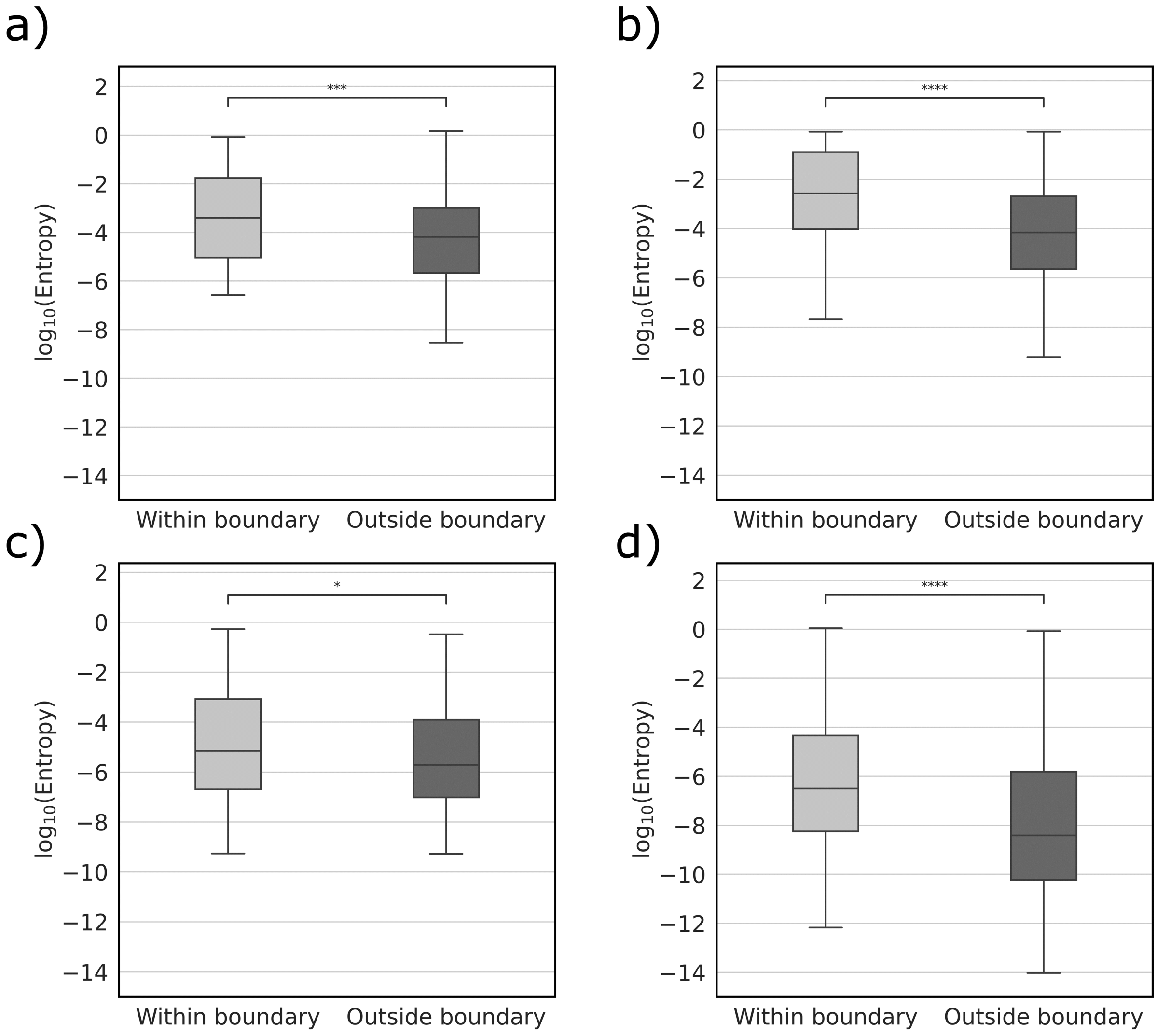


**Figure S10**: Distribution of entropy values for correctly classified CSD validation set sites inside the “twilight zone” and outside for a) SPL/SQP, b) SQP/TBP, c) TET/TBP and d) SQP/OCT RMSD maps shown in Figure 5. p-value annotation legend: ns: p <= 1.00e+00, *: 1.00e-02 < p <= 5.00e-02, **: 1.00e-03 < p <= 1.00e-02, ***: 1.00e-04 < p <= 1.00e-03, ****: p <= 1.00e-04.

**Table S7**: Analysis of statistical correlation between entropy and Continuous Shape Measure (CShM), RMSD and scaled RMSD ($RMSD_{scaled}$). RMSD, $RMSD_{scaled}$ and CShM values are computed against ideal geometry corresponding to each class using pymatgen and cosymlib. $RMSD_{scaled}$ simply refers to RMSD values calculated after scaling all bond lengths to 2 Å, against the ideal geometry. Correlation coefficients ($R_S$) and p-values were obtained using the spearmanr function in scipy.

| **CSD Validation Set** | | | **PDB Validation Set** | | |
|---|---|---|---|---|---|
| **LIN** | | | **LIN** | | |
| | **$R_s$** | **p-value** | | **$R_s$** | **p-value** |
| **CShM** | 0.767968 | 0.002171 | **CShM** | 0.7 | 0.003666 |
| **RMSD** | 0.093407 | 0.7615 | **RMSD** | 0.521429 | 0.046219 |
| **$RMSD_{scaled}$** | 0.747253 | 0.003327 | **$RMSD_{scaled}$** | 0.628571 | 0.01208 |
| | | | | | |
| **TRI** | | | **TRI** | | |
| | **$R_s$** | **p-value** | | **$R_s$** | **p-value** |
| **CShM** | 0.090862 | 0.598176 | **CShM** | -0.27912 | 0.333845 |
| **RMSD** | 0.054311 | 0.753065 | **RMSD** | -0.1956 | 0.50275 |
| **$RMSD_{scaled}$** | 0.099871 | 0.56223 | **$RMSD_{scaled}$** | 0.037363 | 0.899093 |
| | | | | | |
| **TET** | | | **TET** | | |
| | **$R_s$** | **p-value** | | **$R_s$** | **p-value** |
| **CShM** | 0.339679 | 2.58E-07 | **CShM** | 0.381141 | 0.080099 |
| **RMSD** | 0.104171 | 0.124304 | **RMSD** | 0.51214 | 0.01482 |
| **$RMSD_{scaled}$** | 0.406862 | 3.86E-10 | **$RMSD_{scaled}$** | 0.507623 | 0.01588 |
| | | | | | |
| **SPL** | | | **SPL** | | |
| | **$R_s$** | **p-value** | | **$R_s$** | **p-value** |
| **CShM** | 0.465057 | 4.51E-12 | **CShM** | -0.06667 | 0.86469 |
| **RMSD** | 0.445004 | 4.54E-11 | **RMSD** | 0.133333 | 0.732368 |
| **$RMSD_{scaled}$** | 0.520492 | 3.28E-15 | **$RMSD_{scaled}$** | 0.35 | 0.35582 |
| | | | | | |
| **SQP** | | | **SQP** | | |
| | **$R_s$** | **p-value** | | **$R_s$** | **p-value** |
| **CShM** | 0.511715 | 9.77E-14 | **CShM** | 0.456044 | 0.117283 |
| **RMSD** | 0.562628 | 7.77E-17 | **RMSD** | 0.269231 | 0.373733 |
| **$RMSD_{scaled}$** | 0.528613 | 1.04E-14 | **$RMSD_{scaled}$** | 0.67033 | 0.012166 |

| | | | | | |
|---|---|---|---|---|---|
| **TBP** | | | **TBP** | | |
| | **$R_s$** | **p-value** | | **$R_s$** | **p-value** |
| **CShM** | 0.510564 | 1.41E-08 | **CShM** | 0.793407 | 0.000708 |
| **RMSD** | 0.378454 | 4.96E-05 | **RMSD** | 0.797802 | 0.000628 |
| **$RMSD_{scaled}$** | 0.565647 | 1.47E-10 | **$RMSD_{scaled}$** | 0.784615 | 0.00089 |
| | | | | | |
| **OCT** | | | **OCT** | | |
| | **$R_s$** | **p-value** | | **$R_s$** | **p-value** |
| **CShM** | 0.460464 | 3.07E-33 | **CShM** | 0.412281 | 0.079417 |
| **RMSD** | 0.61246 | 7.10E-64 | **RMSD** | 0.457895 | 0.048674 |
| **$RMSD_{scaled}$** | 0.238941 | 2.43E-09 | **$RMSD_{scaled}$** | 0.296491 | 0.21773 |
